# The Involvement of the Laccase Gene *Cglac13* in Mycelial Growth, Germ Tube Development, and the Pathogenicity of *Colletotrichum gloeosporioides* from Mangoes

**DOI:** 10.3390/jof9050503

**Published:** 2023-04-23

**Authors:** Mengting Zhang, Chunli Xiao, Qing Tan, Lingling Dong, Xiaomei Liu, Jinji Pu, He Zhang

**Affiliations:** 1 Key Laboratory of Green Prevention and Control of Tropical Plant Diseases and Pests, Ministry of Education, College of Plant Protection, Hainan University, Haikou 570228, China; 2 Key Laboratory of Integrated Pest Management on Tropical Grops, Ministry of Agriculture and Rural Affairs, Environment and Plant Protection Institute, Chinese Academy of Tropical Agricultural Sciences, Haikou 571101, China; 3 Institute of Tropical Bioscience and Biotechnology, Chinese Academy of Tropical Agricultural Sciences, Haikou 571101, China

**Keywords:** mango, *Colletotrichum gloeosporioides*, laccase, germ tube development, pathogenicity

## Abstract

*Colletotrichum gloeosporioides* is one of the most serious diseases that causes damage to mangoes. Laccase, a copper-containing polyphenol oxidase, has been reported in many species with different functions and activities, and fungal laccase could be closely related to mycelial growth, melanin and appressorium formation, pathogenicity, and so on. Therefore, what is the relationship between laccase and pathogenicity? Do laccase genes have different functions? In this experiment, the knockout mutant and complementary strain of *Cglac13* were obtained through polyethylene glycol (PEG)-mediated protoplast transformation, which then determined the related phenotypes. The results showed that the knockout of *Cglac13* significantly increased the germ tube formation, and the formation rates of appressoria significantly decreased, delaying the mycelial growth and lignin degradation and, ultimately, leading to a significant reduction in the pathogenicity in mango fruit. Furthermore, we observed that *Cglac13* was involved in regulating the formation of germ tubes and appressoria, mycelial growth, lignin degradation, and pathogenicity of *C. gloeosporioides*. This study is the first to report that the function of laccase is related to the formation of germ tubes, and this provides new insights into the pathogenesis of laccase in *C. gloeosporioides*.

## 1. Introduction

The phytopathogen *C. gloeosporioides* is widespread and has infected more than 470 plants [1], such as pear (*Pyrus* spp.) [2], apple (*Malus domestica*) [3], and strawberry (*Fragaria × Ananassa*) [4]. It also infects many tropical fruits, such as mango (*Mangifera indica*) [5], banana (*Musa* spp.) [6], and papaya (*Carica papaya*) [7], as well as some tropical cash crops, such as rubber (*Hevea brasiliensis*) [8] and cassava (*Manihot esculenta*) [9]. Moreover, it results in the occurrence of anthracnose. *C. gloeosporioides* has infected the young fruit of plants with symptoms, such as blackening, irregular shapes, and slightly sunken spots on the fruit. With the gradual increase in the disease spots, the fruit eventually rots [10]. In general, *C. gloeosporioides* is a latent infection of the cuticle or epidermal cells in immature fruits, and it is not revealed until the fruit is ripe [11,12]. Mango anthracnose is one of the significant diseases of both the mango growth and post-harvest periods. 

It is also the most ruinous of all mango diseases and is common in mango-producing areas in the world [10]. When the fruit is planted in high humidity, the incidence of mango anthracnose can be close to 100%, which significantly reduces the economic benefits in various industries and causes significant harm [10,13,14].

In the vast majority of cases, two methods of infection have been identified for the phytopathogenic fungi: one involves mechanical force, and the other is via the secretion of secondary metabolites, such as various cell-wall hydrolases, to promote infection [15]. *C. gloeosporioides* forms germ tubes through the germination of conidia attached to the host surface. Then, the extension top of the germ tubes expands to produce appressoria, and the melanocytes become swollen and form infection nails, invading the host [16]. The germ tube, as one of the infectious structures differentiated during the process of *C. gloeosporioides* infection [17], usually plays an important role. 

Moreover, in *Candida albicans*, the formation of germ tubes is a sign of the transformation of colonized strains to pathogenic strains, and this can also improve their invasiveness and adhesion in the tissues [18]. In the black-spot disease *Dendrobium*, the germ tube of its pathogen invades the dendrobium and secretes toxins and enzymes, destroying the tissues, producing a large number of mycelia, and diffusing to the tissues around the stomata to form a mycelium, which then causes tissue disintegration [19]. *Beauveria brongniartii* penetrates the body wall of the poplar trunk-elephant larva (*Cryptorhynchus lapathi*) in the form of a germ tube or mycelium, and then it produces pathogenic effects on the larva [20]. Therefore, it follows that germ tubes play a role in a variety of pathogens [17,18,19,20].

Laccase, a copper-containing polyphenol oxidase [21], exists in the genome as a gene family and has been reported in several species [22]. At present, fungal laccase has been the most widely studied, and it has a variety of functions related to melanin formation, appressoria penetration, pathogenicity, growth rate, etc. Its role in different pathogenic plant fungi, and even within the same pathogen, has varied [23,24]. Lignin is one of the main components of lignocellulose [25]. It enhances the rigidity and hydrophobicity of plant cell walls [26] and is an important barrier against pests and diseases [27]. Genes such as PAL (phenylalanine ammonia lyase), 4CL (4-coumaric acid-coenzyme ligase), CAD (cinnamon alcohol deoxidase), and COMT (caffeic acid-O-methyltransferase) play important regulatory roles in the lignin-synthesis pathway [28]. 

As a recognized lignin-degrading enzyme, laccase plays an important role in the pathogenesis process [29]. It destroys the lignin structure of plant cell walls, thus, opening the first line of defense for pathogen infection [29]. It also oxidizes phenolic and non-phenolic substances produced by the host during the pathogenesis process and promotes the colonization and expansion of fungi in the host tissues [29]. To date, many studies have reported that laccase is related to the pathogenicity of many plant pathogens. In *Cryphonectria parasitica*, laccase was related to its pathogenicity and the synthesis of melanin [30,31]. 

In *Colletotrichum tuberculosis*, loss pathogenicity occurred after knocking out the laccase gene *LAC2* [32]. In *Setosphaeria turcica*, melanin synthesis was hindered after *StLAC1* mutation, and the pathogenicity was significantly lower than that in the wild-type [33]. *StLAC2* participated in the regulation of lignin degradation and affected the infection process of *S. turcica* [34,35]. In previous studies, we found that laccase participated in the growth regulation of mycelia, which were continuously produced by the germ tubes, forming filamentous or tubular mycelia [36]. 

Nikita et al. found that 11,050 genes in *C. gloeosporioides* were differentially expressed in the process of germ tube formation by whole transcriptome analysis, which encoded cell-wall-degrading enzymes, germination, mycelial growth, host–fungus interaction, and virulence [37]. Among them, pectin lyase, glycosyl hydrolase family 76, and glucose-methanol-choline (GMC) oxidoreductase genes were highly up-regulated during this process [37]. Li et al. confirmed that the expression of the pectin lyase gene was related to laccase in *C. gloeosporioides* [38]. 

As a member of the glycosyl hydrolase family, laccase could slightly inhibit the production of cellulase, according to Hernández et al. [39]. Aryl alcohol oxidase (AAO) belongs to the GMC oxidoreductase superfamily [40]. The products released from its oxidation of benzyl alcohol interacted with laccase to degrade linocellulose and also inhibited the re-polymerization of laccase oxidation products [40,41]. Therefore, we speculated that laccase was indeed related to germ tubes.

Based on this conjecture, we chose the laccase gene family in *C. gloeosporioides* as the research object in order to observe the effect of laccase gene deletion on germ tube formation and reveal the role of the laccase gene in *C. gloeosporioides*. This could provide a basis for analyzing the pathogenic mechanism of *C. gloeosporioides*.

## 2. Materials and Methods

### 2.1. Strains and Culture Conditions

The wild-type strain A2 (No. CATAS-A2) used in this study was provided by Laboratory 405, Institute of Environment and Plant Protection, Chinese Academy of Tropical Agricultural Sciences. It was obtained from the Baodao Xincun Plant Base, Danzhou City, Hainan Province, in May 2006 [36]. 

The strains involved in this experiment were cultured in darkness at 28 °C in PDA (potato 200 g, glucose 20 g, agar 16 g, and adding water to 1 L); PDA containing 0.04% guaiacol (used to observe extracellular laccase enzyme activity); 0.1% H_2_O_2_ (used to analyze the antioxidant capacity of strains), 0.4 M CaCl_2_, 1 M NaCl, and 1 M KCl (used to analyze the effects of hypertonic stress on strains) [42]; and 30.0 g/L saccharose, 15.8 g/L glucose, and 30.0 g/L soluble starch, which were substituted for sucrose in Czapeck (CZA, NaNO_3_ 3 g, K_2_HPO_4_ 1 g, MgSO_4_ 0.5 g, KCl 0.5 g, FeSO_4_ 0.01 g, sucrose 30 g, and added water to 1 L, used to determine the utilization of carbon and nitrogen sources by strains) by equal weight. Similarly, 3.5 g/L L-glutamine, 1.9 g/L ammonium nitrate, and 3.1 g/L ammonium sulfate were substituted for NaNO_3_ in CZA by equal mass. In addition, the colony diameters were measured and statistically analyzed.

### 2.2. Extraction of Plasmids, DNA, RNA, and Expression Levels

The centrifugation of the Escherichia coli strain DH5α solution cultured the knockout and complementary vector in LB medium (tryptone 10 g, yeast extract 5 g, NaCl 10 g, add water to 1 L, and adjust pH of the medium to 7.0) and extracted the plasmid according to the instructions of the Plasmid Mini Kit I (OMEGA, Norcross, GA, USA). We scraped the mycelium on the PDA plate and placed it into a 2 mL round-bottom centrifuge tube (after placing three steel balls into the centrifuge tube in advance). 

Then, we placed it into a grinder to grind and extract DNA according to the steps in the instructions of the Fungal gDNA Kit (OMAGA, Norcross, GA, USA). Next, we placed the treated blades in a prepared mortar and ground the material into a fine powder with liquid nitrogen. We then extracted the total RNA, according to the RNAprep pure plant total RNA extraction kit (centrifugal column type) (TIANGEN, Beijing, China) and observed the imaging with agarose gel electrophoresis. We also used TIANScript cDNA First-Stand Kit (TIANGEN, Beijing, China) to reverse transcribe the cDNA.

For the leaves used in the expression measurement, we first selected the light-green mango leaves that were consistent in the variety (Tainung No. 1) and health status, soaked them in 1% NaClO solution for 5 min, rinsed them with sterile water three times, and then placed them on a sterile operation platform to dry. We gently pricked the leaves with sterilized needles, and then added 3.14 × 10^7^ conidium/mL conidia suspension and moisturizing culture to the wound under dark conditions.

We observed the infection site at 0, 6, 12, 24, 36, 48, and 72 h. Then, we quickly froze the sample in liquid nitrogen and stored it in a refrigerator at −80 °C until use. Each treatment was repeated three times. Using 18s rDNA as the internal reference gene, the relative expressions of *Cglac13* during different time periods of infection were analyzed with primer Cglac13-qPCR-F/R by quantitative real-time PCR.

### 2.3. Primer Design

According to the obtained nucleotide sequence of the *Cglac13* gene, we designed the primers in Primer 5.0 software. Cglac13-qPCR-F/R was used for qRT-PCR to analyze the expression of *Cglac13*, 5Cglac13-MHF/MHR, 3Cglac13-MHF/MHR, and hygB-F/R for knockout vector construction; 5Cglac13-CF/CR, 3Cglac13-CF/CR, and Bar-F/R (F4/R4) for complementary vector construction; and Cglac13H-F/R (F1/R1), H850/H852 (F2/R2), Cglac13 BAR-F/R (F3/R3), and Bar-F/R (F4/R4) for detection of the knockout and complementary mutants (Table 1). 

Among them, the F1/R1 primer pair and the F3/R3 primer pair were designed based on the nucleotide sequence of the knockout vector and the complementary vector, which were specific primers. The former contained the full length of *hygB*, while the latter contained the full length of the two segments of *Cglac13* and *Bar*, and both had upstream and downstream homologous arms. The F2/R2 primer pair and the F4/R4 primer pair were used to detect whether the *hygB* and *Bar* gene fragments had been inserted into the mutant.

### 2.4. Identification of Cglac13

We submitted the amino acid sequence of *Cglac13* to the National Biotechnology Information Center (NCBI) of the United States (https://blast.ncbi.nlm.nih.gov/, accessed on 10 November 2022), and then we used the protein basic local alignment search tool (BLASTP) to analyze the sequence. According to the similarity with the sequence of *Cglac13*, 30 amino acid sequences were screened. 

The 30 amino acid sequences were derived from *Xylaria multiplex* (KAF2965160.1), *Colletotrichum* sp. (KAI8207922.1, KAI8261511.1, KAI8155331.1, and KAI8211189.1), *C. fructicola* (XP_031888153.1), *C. fructicola* (KAF5488340.1), *C. siamense* (KAF4813728.1), *C. gloeosporioides* (XP_045256765.1), *C. asianum* (KAF0317789.1), *C. camelliae* (KAH0431841.1), *C. aenigma* (XP_037174185.1), *C. fructicola* (KAF4888482.1), *C. viniferum* (KAF4899210.1), *C. tropicale* (KAF4820327.1), *Truncatella angustata* (XP_045965109.1), *Podospora comata* (VBB84873.1), *P. anserina* S mat+ (XP_003437543.1), *Xylariales* sp. AK1849 (KAI0131815.1), *Neoarthrinium moseri* (XP_049167532.1), *Thozetella* sp. PMI_491 (KAH8886064.1), *Sordaria macrospora* k-hell (XP_003348049.1), *Neurospora crassa* OR74A (XP_956350.3), *Lomentospora prolificans* (PKS08150.1), *N. tetrasperma* FGSC 2508 (XP_009854786.1), *Neopestalotiopsis* sp. 37M (KAF3022492.1), *N. clavispora* (KAF7540160.1), *Verticillium dahliae* (PNH43866.1), and *V. dahliae* VdLs.17 (XP_009654941.1). 

Furthermore, we compared *Cglac13* with other amino acid sequences containing the 30 amino acid sequences screened from NCBI and the sequences from published studies in this gene family, and then we made 1000 bootstrap replicates. The phylogenetic tree was constructed using the neighbor-joining method in MEGA 11.0, and we provide the details by website (https://itol.embl.de/, accessed on 14 November 2022).

We analyzed the DNA and cDNA sequences of *Cglac13* using DNAssist 1.0 software; then, we uploaded the amino acid sequence of *Cglac13* to the SWISS-MODEL online website (https://swissmodel.expasy.org/, accessed on 25 November 2022), which used a homologous modeling method to predict the three-dimensional structure of the protein. Following a similar protocol, we submitted the sequence of *Cglac13* to the CD-Search tool in NCBI to predict the conserved domain (https://www.ncbi.nlm.nih.gov/, accessed on 29 November 2022). In addition, we constructed a flowchart for the identification of *Cglac13* as shown in Figure S2.

### 2.5. Construction of Plasmids and Acquisition of the Knockout Mutant and Complementary Strain

The resistance gene hygromycin (*hygB*) used in the experiment was derived from the vector pGH14, which was modified based on plasmid pCT74 [43]. The resistance gene glyphosate (*Bar*) was derived from the vector pCAMBIA3300. According to the nucleotide sequence of *Cglac13*, specific primers were designed to amplify the 5’ fragments and 3’ fragments. We used the DNA of a wild-type strain as a template, amplified the 5’ fragments (445) and 3’ fragments (458), and then designed primers based on the sequences of the pGH14 plasmid and amplified *hygB* fragments (1412). Next, we used the In Fusion cloning technique to construct the knockout plasmid [44]. 

For the complementary construct, a fragment consisting of 5’ fragments (2140), the *Bar* gene (552), and 3’ fragments (260) was sequenced using the In Fusion cloning technique to construct the complementary plasmid. All plasmids were propagated in the *Escherichia coli* strain DH5α. In this experiment, the correct knockout and complement vectors were used to obtain knockout mutant and complementary strains through PEG-mediated protoplast transformation. The former was transferred into the wild-type, while the latter was transferred into the mutant and screened in the corresponding resistance plate.

### 2.6. Conidia Germination, Germ Tube Elongation, and Appressorium Formation

For the experiment with conidia, germ tubes, and appressoria, we used the wild-type, mutant *∆Cglac13H* and the complementary strain *C-∆Cglac13H* in 100 mL potato dextrose broth (PDB) and cultured by shaking a conical flask for 3 days at 28 °C, at 180 rpm in the dark. We observed the quantity of conidia on the hemocytometer, and then we collected the conidia, diluted them with sterilized water, and adjusted them to 2 × 10^6^ conidium/mL. The conidia suspension of each strain was placed on a glass slide, and we observed the changes in the conidia at 0, 2, 4, 6, 12, 24, and 36 h with a DM2500 Leica microscope (Leica, Wetzlar, Germany). We used cellSens Dimension 1.11 Software to calculate the conidia germination rate and appressoria formation rate. In addition, we observed the germ tubes of wild-type and mutants at 12 h, statistically analyzed the number and proportion of germ tubes in these two strains and imaged the results.

### 2.7. Pathogenicity Assays

In the pathogenicity observation, we selected fruits (firmness: 7 N·cm^−2^; color: L: 90.69; a: 4.83; b: 33.31; and TSS: 12.8%) that were consistent in the variety (Tainung No. 1) and health status, with a smooth surface, physiological maturity, and no damage. The specific disinfection method was the same as in Section 2.2. We used it to observe and measure the changes in pathogenicity after the *Cglac13* knockout. The treatment method was similar to that of the leaves, with the exception of inoculating each strain cake on the wound. We inoculated the wild-type, mutant *∆Cglac13H*, and complementary strain *C-∆Cglac13H* with the same cultivation time on the mango fruit, which were treated according to two groups, wounded and unwounded. 

After inoculation, the fruits were stored at 28 °C, RH ≥ 95% for 3 days, and then we observed and measured the disease spots. We cut the infected position of the mango peel on the third day. We used the Lignin Content Detection Kit (Solarbio, Beijing, China) and QuantStudio 6 Flex (Applied Biosystems, Waltham, MA, USA) to determine the lignin content and the expression of genes related to lignin biosynthesis of the wild-type, mutant *∆Cglac13H*, and complementary strain *C-∆Cglac13H*.

### 2.8. Statistical Analysis

All the data were processed in IBM SPSS Statistics 26, and we used the One-Way ANOVA test for analysis. We assumed equal variance by the selected Duncan’s method (*p* < 0.01). In this study, uppercase letters indicate an extremely significant difference (*p* < 0.01), and lowercase letters indicate a significant difference (*p* < 0.05).

## 3. Results

### 3.1. Identification and Phylogenetic Analysis of Cglac13

**Figure 1:**
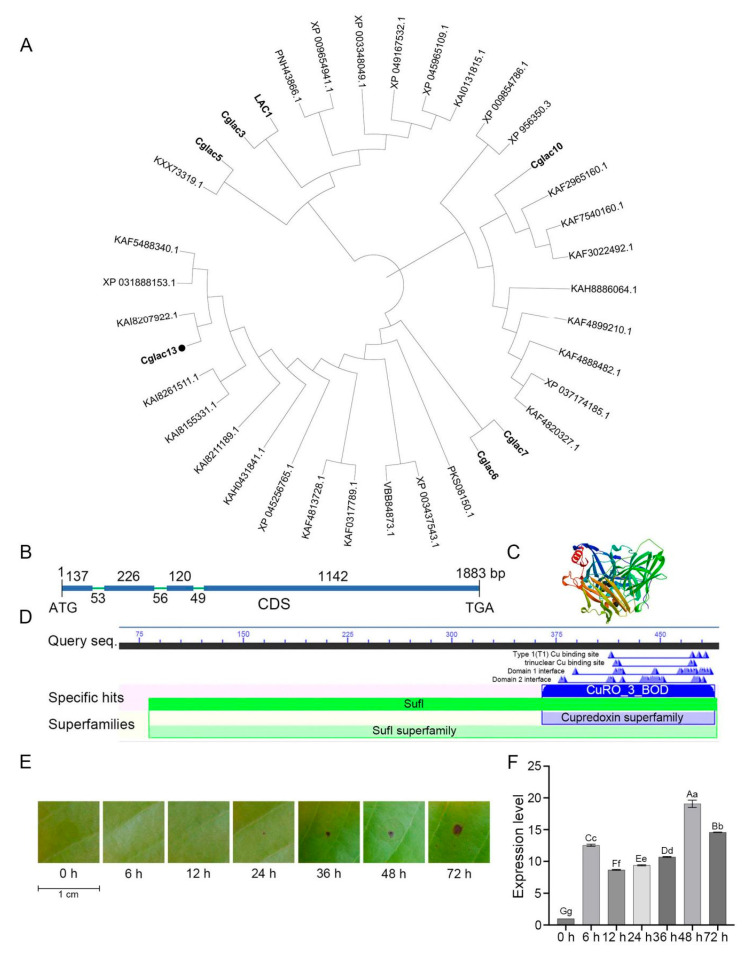
Identification and phylogenetic analysis of *Cglac13*. (**A**) Phylogenetic tree of *Cglac13* based on the neighbor-joining method. *Cglac13* has a circle mark, and the genes of the same laccase gene family are shown in bold type. (**B**) The CDS structural pattern diagram of the *Cglac13* gene. The full-length DNA sequence of the *Cglac13* gene was 1883 bp, and its cDNA sequence was 1725 bp, including four exons and three introns. (**C**) The protein 3D-Structure of *Cglac13* as generated by the SWISS-MODEL website. (**D**) Analysis of the conserved domain of *Cglac13*: We used the CD-search tool in NCBI to predict the conserved domain of *Cglac13*. (**E**) The process of *C. gloeosporioides* infecting leaves at 0, 6, 12, 24, 36, 48, and 72 h. We gently pricked the leaves with sterilized needles and dropped 3.14 × 10^7^ conidium/mL conidia suspension at the wound to moisturize and cultivate in the dark. (**F**) Statistical analysis of the expression of *Cglac13* at 0, 6, 12, 24, 36, 48, and 72 h during *C. gloeosporioides* infection of the leaves (*p* < 0.01). *Cglac13* remained highly expressed throughout the whole process of leaf infection, with the relative expression of *Cglac13* being more than eight-times higher at all times points, as compared to the expression at 0 h, and the highest was at 48 h. (A, B, C, D, E, F and G in the figure indicate extremely significant differences, while a, b, c, d, e, f and g indicate significant differences.)

The phylogenetic analysis results indicated that the Cglac13 protein and *Colletotrichum* spp. bilirubin oxidase protein KAI8207922.1 (a copper-containing polyphenol oxidase) were clustered on the same branch. *Cglac13* is a homologous gene of the laccase genes *LAC1*, *Cglac3*, *Cglac5*, *Cglac6*, *Cglac7*, and *Cglac10*, which was studied at an earlier stage (Figure 1A). DNAssist showed that the total length of *Cglac13* was 1883 bp, the length of CDS was 1725 bp, and there were four exons and three introns in *Cglac13* (Figure 1B). *Cglac13*, with multiple Cu binding sites and a CuRO-3-BOD conserved domain, belonged to the cupredoxin superfamily (Figure 1C,D). 

These results indicated that *Cglac13* is a copper-containing polyphenol oxidase. The expression levels of *Cglac13* in the mango leaves at different time points of *C. gloeosporioides* infection were analyzed by qRT-PCR (Figure 1E,F). The results showed that *Cglac13* remained highly expressed throughout the infection process of *C. gloeosporioides*. As compared to the observations at 0 h, the relative expression levels of *Cglac13* were from 8.66 to 19.07 of the study period. In summary, the *Cglac13* gene had a positive response to the infection of mango by *C. gloeosporioides*.

### 3.2. Validation of Gene Knockout Mutant and Complementary Strain

**Figure 2:**
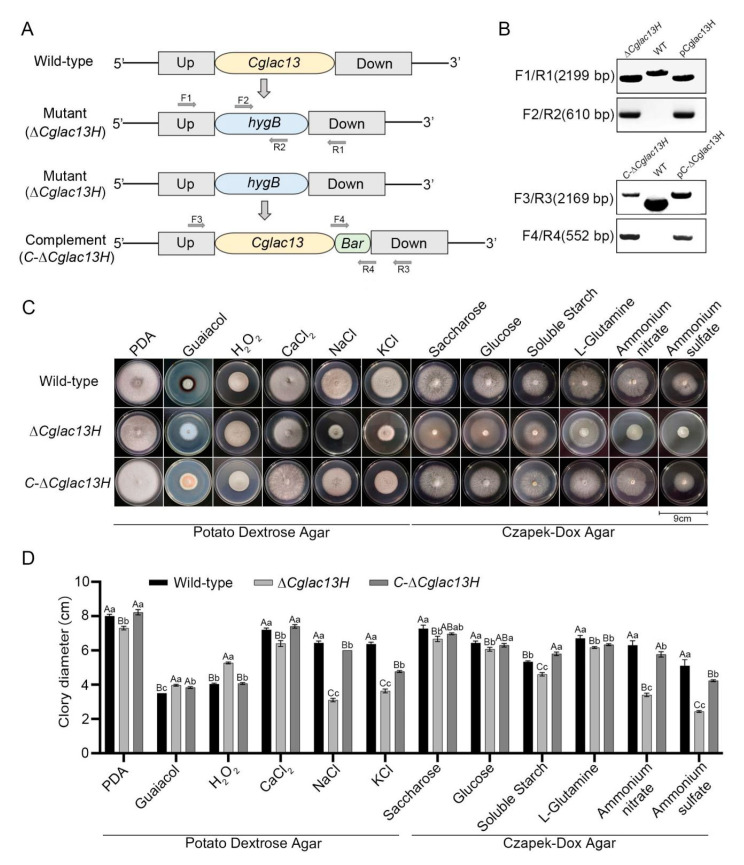
Generation of *Cglac13* knockout and complementary strains. (**A**) The mutant *∆Cglac13H* comprised *Cglac13*-flanking sequences and *hygB* and was designed to replace the *Cglac13* gene by homologous recombination. The complementary strain *C-∆Cglac13H*, based on the mutant *∆Cglac13H*, replaced the *Cglac13* sequence and *Bar* with *hygB* through homologous recombination. (**B**) PCR verification of primers F1/R1, F2/R2, F3/R3, and F4/R4. The electropherogram of primers F1/R1 and F2/R2: lane 1: mutant *∆Cglac13H*, lane 2: wild-type, and lane 3: pCglac13H; the electropherogram of primers F3/R3 and F4/R4: lane 1: the complementary strain *C-∆Cglac13H*, lane 2: wild-type, and lane 3: pC-*∆Cglac13H*. (**C**) The wild-type, mutant *∆Cglac13H* and the complementary strain *C-∆Cglac13H* were inoculated on culture media under different conditions. a: PDA, b: Guaiacol + PDA, c: 0.1% H_2_O_2_ + PDA, d: CaCl_2_ + PDA, e: NaCl + PDA, f: KCl + PDA, g: Saccharose + CZA, h: Glucose + CZA, i: Soluble Starch + CZA, j: L-Glutamine + CZA, k: Ammonium nitrate + CZA, and l: Ammonium sulfate + CZA. (**D**) Statistical analysis of the colony diameter variations on culture media under different conditions in (**C**) (cm) (*p* < 0.01). (A, B, and C in the figure indicate extremely significant differences, while a, b, and c indicate significant differences.)

The knockout and complement vectors of *Cglac13* were constructed by homologous recombination (Figure 2A), and the knockout and complement transformants were obtained by polyethylene glycol (PEG)-mediated protoplast transformation. We used two primer pairs, F1/R1 and F2/R2, for the PCR amplification in order to detect the knockout mutant of the transformants. The F1/R1 primer pair amplified specific 2199 bp bands for the knockout mutant and knockout vector pCglac13H, which were lower than the wild-type bands, indicating that the *Cglac13* gene had been successfully replaced by *hygB* (Figure 2B). 

At the same time, a specific bright band at 610 bp in the knockout mutant and the knockout vector pCglac13H was found in the amplified transformants of the F2/R2 primer pair, indicating that the *hygB* gene was successfully transferred to the transformants. Similarly, the PCR amplification of primer pairs F3/R3 and F4/R4 was used to detect the complementary transformants (Figure 2B), and the complete electrophoretogram of these four pairs of primers is shown in Figure S1. In summary, the knockout mutant and complementary strain of *Cglac13* were successfully obtained, and they were named *∆Cglac13H* and *C-∆Cglac13H*, respectively.

The inoculated wild-type, the knockout mutant *∆Cglac13H*, and the complementary strain *C-∆Cglac13H* were added to the culture media under different conditions to observe the mycelial growth in each strain (Figure 2C,D). In the PDA plate culture containing guaiacol, there was an obvious red circle in the wild-type and complementary colonies but not in the knockout mutant (Figure 2C). The results showed that, in the PDA media containing NaCl and KCl and in the CZA media containing ammonium nitrate and ammonium sulfate, the colony diameters of the knockout mutant, wild-type, and complementary strain had the largest differences, as compared to those in other conditions, and the colony diameters decreased by 51.81%, 42.93%, 46.03%, and 52.29%, respectively (Figure 2C,D). 

In the PDA medium containing 0.1% H_2_O_2_, the colony diameter of the mutant significantly increased (Figure 2C,D). In addition, in the basic PDA medium, the PDA medium containing CaCl_2_ and the other CZA media containing carbon and nitrogen sources, the colony diameter of mutant also decreased significantly compared to the wild-type.

### 3.3. Cglac13 Involves Conidial Germination and Appressoria Formation

**Figure 3:**
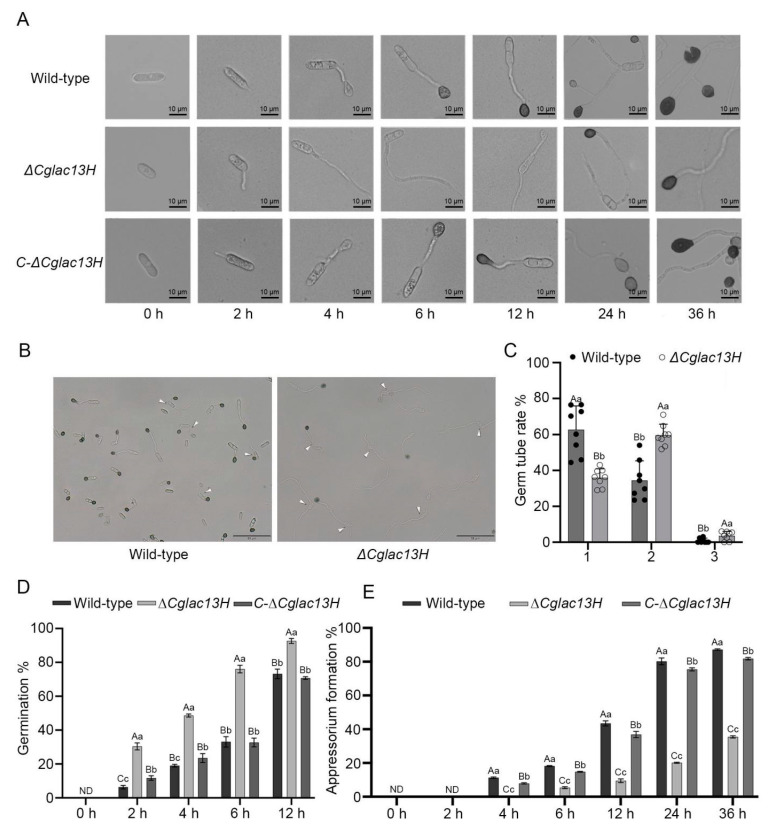
Growth and development of *Cglac13*. (**A**) The conidia suspension of the wild-type, mutant *∆Cglac13H*, and complementary strain *C-∆Cglac13H* were used to observe the conidia morphology of each strain under a microscope at 0, 2, 4, 6, 12, 24, and 36 h. (**B**) The number of germ tubes at 12 h: wild-type (**left**) and mutant (**right**). The conidia that produced two or more germ tubes are marked by a triangle symbol. Additionally, those with inconspicuous germ tube lengths are not included. (**C**) Statistical analysis of the proportion of wild-type and mutant conidia produced one, two, or three germ tubes to the total germination conidia at 12 h (*p* < 0.01). (**D**) Statistical analysis of germination rate of conidia at 0, 2, 4, 6, and 12 h (*p* < 0.01). (**E**) Statistical analysis of appressorium formation rate at 0, 2, 4, 6, 12, 24, and 36 h (*p* < 0.01). (A, B, and C in the figure indicate extremely significant differences, while a, b, and c indicate significant differences.)

We collected the conidia of the wild-type, knockout mutant *∆Cglac13H*, and complementary strain *C-∆Cglac13H*. The conidia of the knockout mutant *∆Cglac13H* germinated normally, produced germ tubes, and formed melanized appressoria normally. The germ tubes of the mutant were thinner and longer than those in the wild-type (Figure 3A). The number and percentage of germ tubes in the mutant *∆Cglac13H* and the wild-type were observed and counted at 12 h (Figure 3B,C). Knockout mutant *∆Cglac13H* and wild-type conidia germinated one, two, or three germ tubes. 

As compared to the wild-type, the percentage of germination from two to three germ tubes was significantly higher in the knockout mutant (Figure 3C). The percentages of the knockout mutant and wild-type that germinated two germ tubes were 59.44% and 34.33%, respectively (Figure 3C). During the process of germination, we observed that the germination rate of *∆Cglac13H* was significantly higher than those of the wild-type and complementary strain (Figure 3D). We observed the formation of appressoria under a microscope (Figure 3E). 

We found that appressoria began to form in the wild-type and complementary strain after 4 h, and the melanized, mature appressoria appeared 6 h afterwards. The appressorium formation rate in the wild-type reached 18.17%, while the germ tube of the knockout mutant continued to expand at this time, and few appressoria were formed. After 24 h, the appressorium formation rate of the mutant reached 20.13%. Then, the appressoria of the mutant gradually blackened and matured at 36 h. At this moment, the appressorium formation rate of the wild-type reached approximately 87.20%. The knockout of the *Cglac13* gene delayed the appressorium formation of *C. gloeosporioides* and significantly reduced the formation rate (Figure 3A,E). The results showed that *Cglac13* regulated the conidia germination, the germ tube development, and appressorium formation in *C. gloeosporioides*.

### 3.4. Cglac13 Is Required for Pathogenicity

**Figure 4:**
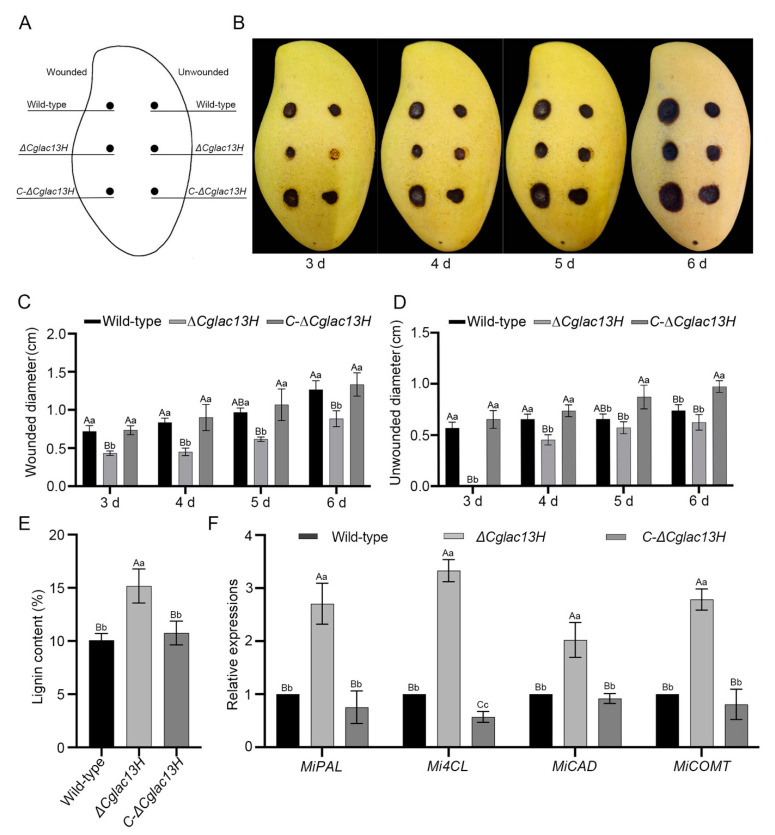
Pathogenicity determination of *Cglac13*. (**A**) This figure mirrors the patterns in (**B**): as shown in the illustration, the left side is the stab wound treatment, and the right side is the non-stab wound treatment. The circle shows the inoculated strains, which are wild-type, mutant, and complementary strain from top to bottom. (**B**) Virulence assays on mango fruits of the wild-type, mutant *∆Cglac13H*, and complementary strain *C-∆Cglac13H*. The picture shows the disease spots on the 3rd, 4th, 5th and 6th days. (**C**) Statistical analysis of the spot diameter in wild-type, mutant *∆Cglac13H*, and complementary strain *C-∆Cglac13H*, with stab inoculation (cm) (*p* < 0.01). (**D**) Statistical analysis of the spot diameter of wild-type, mutant *ΔCglac13H*, and complementary strain *C-∆Cglac13H*, without stab inoculation (cm) (*p* < 0.01). (**E**) The percentage content of lignin in mango peels inoculated in wild-type, mutant *∆Cglac13H*, and complementary strain *C-∆Cglac13H*, on the third day (*p* < 0.01). (**F**) The relative expression of genes related to lignin synthesis in mango peel inoculated in the wild-type, mutant, and complementary strains, on the third day (*p* < 0.01). In this experiment, *MiActin* (JF737036.1) [45] was used as the reference gene, and the fruit peel inoculated in the wild-type was the control. We calculated the relative expression levels of *MiPAL* [46], *Mi4CL* (XM_044609362.1) [47], *MiCAD* (XM_044654805.1) [47], and *MiCOMT* (XM_044626972.1) on fruit peels in the inoculated mutant and complementary strain. (A, B, and C in the figure indicate extremely significant differences, while a, b, and c indicate significant differences.)

Concerning the plant pathogenic fungus, the pathogenicities of wild-type, mutant *∆Cglac13H*, and complementary strain *C-∆Cglac13H* on the physiological maturity of the mango fruits were studied (Figure 4A). The wild-type and complementary strain inoculated with stab wounds had significantly more damage to the pericarp tissue, while the disease spots inoculated with the knockout mutant were significantly smaller (Figure 4B). In the puncture inoculation, the disease spot diameters of the knockout mutant were significantly lower than those in the wild-type (Figure 4C). There were no significant differences in the diameters of the disease spot between the complementary strain and wild-type. 

Without the puncture inoculation, the disease spot diameters of the knockout mutant were significantly smaller than those in the wild-type and complementary strain (Figure 4D). As a recognized lignin-degrading enzyme, laccase was used to study the lignin content and the expression levels of the lignin-synthesis pathway and related genes in the mango peels inoculated on the third day. The lignin content of the knockout mutant was higher than in the wild-type and complementary strain with extremely significant differences (Figure 4E). At the same time, the relative expression levels of the related genes in the mutant were also significantly higher than in the wild-type and complementary strain (Figure 4F). The results suggest that *Cglac13* participated in the degradation of lignin and significantly affected the pathogenicity of *C. gloeosporioides* with mangoes.

## 4. Discussion

Previous studies showed that the vegetative growth of hyphae is the prerequisite for producing conidia and infecting host cells in *Magnaporthe oryzae* [48]. The results of this experiment showed that the colony diameter of the knockout mutant *∆Cglac13H* was significantly different from that in the wild-type in different media. With the exception of the significant increase in the colony diameter on the guaiacol and H_2_O_2_ plates, the others were significantly decreased. 

Among them, the difference in the colony diameter was the most obvious on the plate with KCl, NaCl, ammonium nitrate, and ammonium sulfate as substrates, indicating that, when compared to other hypertonic conditions, the tolerance of mutant *∆Cglac13H* to KCl and NaCl was significantly lower, and the utilization rate of the nitrogen sources, such as ammonium nitrate and ammonium sulfate, was significantly reduced. The research results of Cañero et al. are consistent with the results of this study, where the laccase gene *lcc3* of *Fusarium oxysporum* was involved in regulating the nutrient growth, carbon source metabolism, and oxidative stress [49]. In addition, the knockout of *Shlac* in *Scleromitrula shiraiana* had similar results [50]. 

Fungal laccase participated in regulating the morphogenesis, growth, and development of pathogens [29]. Compared to our previous research, the mycelial growth results of *LAC1* [36] and *Cglac13* on PDA are consistent. In the relevant stress experiments, *LAC1* [36] and *Cglac10* are similar to *Cglac13*. In addition, in the experiment on carbon and nitrogen source utilization, the results of *LAC1* [36], *Cglac10*, and *Cglac13* are consistent. Therefore, we conclude that *Cglac13* is involved in the mycelial growth, the stress response, and the utilization of carbon and nitrogen sources in *C. gloeosporioides*.

Plant pathogen fungi, such as *M. oryzae* and *C. gloeosporioides*, could produce turgor through the appressoria, form a penetration peg, and invade the host, thus, causing plant disease [15,51]. We found that, compared to the wild-type, the appressorium formation rate and pathogenicity of the mutant *∆Cglac13H* significantly decreased, which was consistent with the results of *LAC1* and *Cglac10* [36,52]. In addition, the knockout of the *CgHOS2* [53], *CgHSF1* [8], *CgGa1* [54], *CgCPS1* [55], *CgOPT2* [56], and *CgEnd3* [57] genes in *C. gloeosporioides* had the same results. This illustrates that *Cglac13* significantly affected the appressorium formation and pathogenicity in *C. gloeosporioides*.

A germ tube is one of the infection structures of pathogenic plant fungi. In *Metarhizium anisopliae*, the virulent *pr1* gene regulates appressorium development but does not regulate conidia germination and germ tube elongation [58,59]. In *Verticillium dahliae*, the knockout *CPMO-1* and *CPMO-2* genes reduced the number of microsclerotia and produced more germ tubes [60]. In *C. gloeosporioides*, the *CgHOS2* [53] and *PniiA-Cghsf1* [8] genes regulated the lengths of the germ tubes and the morphology of appressoria [8,53]. 

In an experiment on *CgHSF1*, Xuesheng Gao et al. found that the mutant *PniiA-Cghsf1* weakened melanin synthesis by affecting the transcription of melanin-related genes and the formation of germ tubes and appressoria, thereby reducing the pathogenicity [8]. In contrast to these studies, the number of germ tubes after the deletion of *Cglac13* was statistically analyzed in this experiment. We calculated the proportions of the wild-type, mutant *∆Cglac13H*, and complementary strain *C-∆Cglac13H* producing one, two, or three germ tubes at 12 h. 

**Figure 5:**
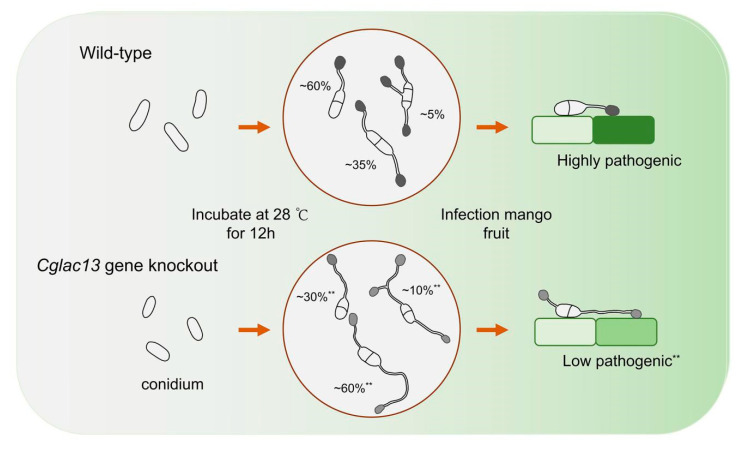
Schematic model for the process variation of *C. gloeosporioides* infection in mango fruit caused by a *Cglac13* gene knockout. The percentage refers to the proportion of one, two, or three germ tubes of conidium germination of the wild-type and *Cglac13* gene knockout compared to the total number of germinated tubes. For the pathogenicity part, we used the type with the largest number of germination tubes in the wild-type and *Cglac13* gene knockout conidia as examples. The number of conidium germinations with one germ tube in the wild-type was the largest, and the number of conidium germinations with two germ tubes in the *Cglac13* gene knockout was the largest. “**” indicates an extremely significant difference compared to the wild-type (*p* < 0.01).

We found that the *Cglac13* gene regulated both the number and length of the germ tubes (Figure 5), and, when compared to the knockout mutant, the wild-type showed decreases in these values by 42.24% and 63.10%, respectively. The results showed that *Cglac13*, as a member of the laccase gene family, was involved in the regulation of germ tube development. 

The infection of the pathogen in the host is affected by many factors, among which temperature and relative humidity are important environmental factors [61]. Inappropriate temperature and relative humidity conditions can lead to the failure of conidia germination and delay the formation of appressoria. In this experiment, we speculated that the decrease in pathogenicity in the knockout mutant would be related to their formation of germ tubes, as this would promote the elongation of germ tubes and result in a decrease in the ability to form adherents and a delay in their formation time as well as a reduction in the success rate of the pathogen infection and, thus, the pathogenicity.

## 5. Conclusions

In this study, knockout mutant and complementary strains of *Cglac13* were obtained through PEG-mediated protoplast transformation. Our results showed that *Cglac13* was involved in regulating the formation of germ tubes and appressoria, mycelial growth, lignin degradation, and pathogenicity in *C. gloeosporioides*. For the first time, we proved that the laccase gene was related to the formation of germ tubes, and we provided new insights into the pathogenesis of laccase from *C. gloeosporioides*. In addition, we speculated that laccase could also be related to the formation of germ tubes in filamentous fungi other than *C. gloeosporioides*.

## Figures and Tables

**Table 1:** The primers used in this study. All primers were designed by Primer 5.0 software, and the complete sequence of each primer is shown in Text S1.

Primer Name	Sequence (5′-3′)	Expected Length/bp
Cglac13-qPCR-F	CCACTGCCACAATCTTAT	178
Cglac13-qPCR-R	TTGCACCTTCTGCACAAC	
5Cglac13-MHF	AACGAAAGCCAGCGACAA	445
5Cglac13-MHR	CTGTACTCAGGACTCAGCCAGT	
3Cglac13-MHF	ATGGAGCAGGTTGATGAGATT	458
3Cglac13-MHR	ATGGGAAGGAGGAGTGGG	
hygB-F	AACTGGTTCCCGGTCGGC	1412
hygB-R	AACTGATATTGAAGGAGCATTTTTT	
5Cglac13-CF	TCTAGACAGACACGCAGC	2140
5Cglac13-CR	AAATCTTACACCCCCAAT	
3Cglac13-CF	TAAGATTACGGTGATGGC	260
3Cglac13-CR	GATAGATTCTGATGGGGA	
F1-Cglac13H	CGGGAGCACAACAGCAAT	2199 (Mutant) 2554 (Wild-type) 2199 (pCglac13H)
R1-Cglac13H	GGAGGAGTGGGTTAGCGTAG	
F2-H850	AACTCACCGCGACGTCTGTC	610 (Mutant) 0 (Wild-type) 610 (pCglac13H)
R2-H852	TTGTCCGTCAGGACATTGTT	
F3-Cglac13 BAR	GAGTGCCCGCTCCTGTGGTA	2169 (Complementary strain) 1618 (Wild-type) 0 (Mutant)
R3-Cglac13 BAR	GATTCTGATGGGGAAATTTG	
F4-Bar	TCAAATCTCGGTGACG	552 (Complementary strain) 0 (Mutant; Wild-type)
R4-Bar	ATGAGCCCAGAACGACGC	
MiActin-qPCR-F	GTTTCCCAGTATTGTGGGTAGG	167
MiActin-qPCR-R	AGATCTTTTCCATATCATCCCAGTT	
MiPAL-qPCR-F	GTCGCATAGGAGAACGAAGC	204
MiPAL-qPCR-R	AACTTGGTGATGGCTTCCAG	
Mi4CL-qPCR-F	GAATACGCTTTCTCTCTCAGAG	188
Mi4CL-qPCR-R	GAGTTGGGAGAGAGTACAAATG	
MiCAD-qPCR-F	CGGCAAGATTACACCTTACACAT	170
MiCAD-qPCR-R	TAACCACCCCAGTAATTTCATGC	
MiCOMT-qPCR-F	GGCAAAGATCCCAGATTCAA	228
MiCOMT-qPCR-R	CAAAGATGGAGCATCGTCAA	

## Data Availability

The data that support the findings of this study are available from the corresponding author upon reasonable request.

## Supplementary Materials

The following supporting information can be downloaded at: https://www.mdpi.com/article/10.3390/jof9050503/s1, Figure S1: The electropherogram of primers F1/R1, F2/R2, F3/R3, and F4/R4; Figure S2: The flowchart of *Cglac13* identification; Text S1: Primer sequence of *Cglac13*.

## References

[1] Siddiqui Y., Ali A. (2014). Chapter 11—*Colletotrichum gloeosporioides* (Anthracnose). Postharvest Decay.

[2] Tashiro N., Manabe K., Ide Y. (2012). Emergence and frequency of highly benzimidazole-resistant *Colletotrichum gloeosporioides*, pathogen of Japanese pear anthracnose, after discontinued use of benzimidazole. J. Gen. Plant Pathol..

[3] Azeem M., Zhou Z., Zhang J., Khaskheli M.I., Rui J.Z., Khaskheli A.J., Ali S. (2021). Pathogenic and biological characterisation of T-DNA insertional mutants of a *Colletotrichum gloeosporioides* casual organism of apple anthracnose. Hortic. Sci..

[4] Wu J., Hu S., Ye B., Hu X., Xiao W., Yu H., Zhang C. (2022). Diversity and resistance to thiophanate-methyl of *Colletotrichum* spp. in strawberry nursery and the development of rapid detection ssing LAMP method. Agronomy.

[5] Kaviyarasi M., Kamalakannan A., Rajendran L., Rajesh S., Kavino M., Swarna L.K.R., Shajith B.J. (2022). Morphological and molecular characterization of *Colletotrichum gloeosporioides* causing mango anthracnose. Int. J. Plant Soil. Sci..

[6] Riera N., Ramirez V.D., Barriga M.N., Alvarez S.J., Herrera K., Ruales C., Leon R.A. (2019). First report of banana anthracnose caused by *Colletotrichum gloeosporioides* in Ecuador. Plant Dis..

[7] Vieira W.A.d.S., Veloso J.S., Silva A.C.d., Nunes A.d.S., Doyle V.P., Castlebury L.A., Câmara M.P.S. (2022). Elucidating the *Colletotrichum* spp. diversity responsible for papaya anthracnose in Brazil. Fungal Biol..

[8] Gao X., Wang Q., Feng Q., Zhang B., He C., Luo H., An B. (2022). Heat shock transcription factor *CgHSF1* is required for melanin biosynthesis, appressorium formation, and pathogenicity in *Colletotrichum gloeosporioides*. J. Fungi.

[9] Sangpueak R., Phansak P., Thumanu K., Siriwong S., Wongkaew S., Buensanteai N. (2021). Effect of salicylic acid formulations on induced plant defense against cassava anthracnose disease. Plant Pathol. J..

[10] Sharma A., Sharma I.M., Sharma M., Sharma K., Sharma A. (2021). Effectiveness of fungal, bacterial and yeast antagonists for management of mango anthracnose (*Colletotrichum gloeosporioides*). Egypt J. Biol. Pest Control.

[11] Silva D.D., Crous P.W., Ades P.K., Hyde K.D., Taylor P. (2016). Life styles of *Colletotrichum* species and implications for plant biosecurity. Fungal Biol. Rev..

[12] Ren D., Wang T., Zhou G., Ren W., Duan X., Gao L., Chen J., Xu L., Zhu P. (2022). Ethylene promotes expression of the appressorium and pathogenicity related genes via GPCR and MAPK dependent manners in *Colletotrichum gloeosporioides*. J. Fungi.

[13] Reyes-Perez J.J., Hernandez-Montiel L.G., Vero S., Noa-Carrazana J.C., Quiñones-Aguilar E.E., Rincón-Enríquez G. (2019). Postharvest biocontrol of *Colletotrichum gloeosporioides* on mango using the marine bacterium *Stenotrophomonas rhizophila* and its possible mechanisms of action. Food Sci. Technol..

[14] Arauz L.F. (2000). Mango anthracnose: Economic impact and current options for integrated managaement. Plant Dis..

[15] Yu H., Lan J., Liu L. (2022). The process of infection of mango by *Colletotrichum gloeosporum* and the histopathological study of its host. Chin. J. Trop. Crops.

[16] Lan J. (2012). Study on the Taxonomy of Colletotrichum Corda et al. in China and Biological Peculiarity of *Colletotrichum gloeosporioides* (Penz.) Sacc. in Mango. Ph.D. Dissertation.

[17] Mcdowell J.M. (2013). Genomic and transcriptomic insights into lifestyle transitions of ahemi-biotrophic fungal pathogen. New Phytol..

[18] Philipp J., Mischo C.E., Gubesh G., Christian S., Becker S.L., Bernhard H., Karin J., Markus B. (2020). *Candida albicans* adhesion to central venous catheters: Impact of blood plasma-driven germ tube formation and pathogen-derived adhesins. Virulence.

[19] Zhang J., Zheng X. (2004). Identification of the pathogen of *Dendrobium candidum* black spot and cytological study on the infection process. J. Plant Pathol..

[20] Cao Q., Chi D., Yu J., Ran Y. (2015). Scanning electron microscope and transmission electron microscope observation of the body wall of *Beauveria bassiana* infecting the larvae of *Dendrolimus poplar*. J. Beijing For. Univ..

[21] Nakamura K., GO N. (2005). Function and molecular evolution of multicopper blue proteins. Cell Mol. Life Sci..

[22] Kües U., Rühl M. (2011). Multiple multi-copper oxidase gene families in basidiomycete- what for. Curr. Genom..

[23] Liu N., Qu Q., Li L., Pang Q., Liu J., Zhang Y., Cao Z., Dong J. (2019). Identification and expression pattern of laccase like copper oxidase from *Fusarium graminearum*. J. Plant Pathol..

[24] Chung H.J., Kwon B.R., Kim J.M., Park S.M., Park J.K., Cha B.J., Yang M.S., Kim D.H. (2008). A tannic acid-inducible and hypoviral-regulated laccase3 contributes to the virulence of the chestnut blight fungus *Cryphonectria parasitica*. Mol. Plant Microbe Interact..

[25] Lankiewicz T.S., Choudhary H., Gao Y., Amer B., Lillington S.P., Leggieri P.A., Brown J.L., Swift C.L., Lipzen A., Na H. (2023). Lignin deconstruction by anaerobic fungi. Nat. Microbiol..

[26] Schuetz M., Benske A., Smith R.A., Watanabe Y., Tobimatsu Y., Ralph J., Demura T., Ellis B., Samuels A.L. (2014). Laccases direct lignification in the discrete secondary cell wall domains of protoxylem. Plant Physiol..

[27] Ithal N., Recknor J., Nettleton D., Maier T., Baum T.J., Mitchum M.G. (2007). Developmental transcript profiling of cyst nematode feeding cells in soybean roots. Mol. Plant Microbe Interact..

[28] Liu Q., Luo L., Zheng L. (2018). Lignins: Biosynthesis and Biological Functions in Plants. Int. J. Mol. Sci..

[29] Liu N., Jia H., Shen S., Cao Z., Dong J. (2020). Fungal laccase: Various biological functions and complex natural substrate. J. Agric. Biotechnol..

[30] Choi G.H., Larson T.G., Nuss D.L. (1992). Molecular analysis of the laccase gene from the chestnut blight fungus and selective suppression of its expression in an isogenic hypovirulent strain. Mol. Plant Microbe Interact..

[31] Valeru S.P., Rompikuntal P.K., Ishikawa T., Vaitkevicius K., Sjöling A., Dolganov N., Zhu J., Schoolnik G., Wai S.N. (2009). Role of melanin pigment in expression of Vibrio cholerae virulence factors. Infect. Immun..

[32] Lin S.Y., Okuda S., Ikeda K., Okuno T., Takano Y. (2012). *LAC2* encoding a secreted laccase is involved in appressorial melanization and conidial pigmentation in *Colletotrichum orbiculare*. Mol. Plant Microbe Interact..

[33] Zhan X. (2011). Function Analysis of Laccase Genes (StLAC) in Melanin Biosynthesis Pathway of Setosphaeria turcica.

[34] Ma S., Liu N., Jia H., Dai D., Xu M., Cao Z., Dong J. (2016). Analysis and Expression of Laccase Gene *Stlac2* in *Setosphaeria turcica*. Chin. Agric. Sci..

[35] Jia H., Meng Q., Li Z., Gong X., Cang J., Hao Z., Cao Z., Dong J. (2015). Localization of Melanin Biosynthesis Enzyme Genes in the Genome and Expression Pattern Analysis of *Setosphaeria turcica*. Chin. Agric. Sci..

[36] Wei Y., Pu J., Zhang H., Liu Y., Zhou F., Zhang K., Liu X. (2017). The laccase gene (*LAC1*) is essential for *Colletotrichum gloeosporioides* development and virulence on mango leaves and fruits. Physiol. Mol. Plant Pathol..

[37] Mehta N., Patil R., Baghela A. (2021). Differential physiological prerequisites and gene expression profiles of conidial anastomosis tube and germ tube formation in *Colletotrichum gloeosporioides*. J. Fungi.

[38] Li H., Zhong C., Wu Q., Zhang Y., Zhang H., Pu J., Liu X. (2018). Sequence characteristics of three pectin lyase genes of mango anthracnose and analysis of their influence by laccase gene *Lac1*. Chin. J. Trop. Crops.

[39] Hernández C., Farnet D.S.A.M., Ziarelli F., Perraud G.I., Gutiérrez R.B., García P.J.A., Alarcón E. (2017). Laccase induction by synthetic dyes in *Pycnoporus sanguineus* and their possible use for sugar cane bagasse delignification. Appl. Microbiol. Biot..

[40] Goswami P., Chinnadayyala S.S.R., Chakraborty M., Kumar A.K., Kakoti A. (2013). An overview on alcohol oxidases and their potential applications. Appl. Microbiol. Biot..

[41] Yan J., Tong Z., Liu Y., Zhang L., Zhang Y., Xie B. (2018). Sequence characteristics and differential expression of volvariella volvacea aromatic alcohol oxidase gene vvaao1. Biotechnol. Bull..

[42] Gu S. (2007). Cloning and Functional Analysis of the STK Genes that Regulates the Growth, Development and Pathogenicity of Setosphaeria turcica.

[43] Lorang J.M., Tuori R.P., Martinez J.P., Sawyer T.L., Redman R.S., Rollins J.A., Wolpert T.J., Johnson K.B., Rodriguez R.J., Dickman M.B. (2001). Green fluorescent protein is lighting up fungal biology. Appl. Environ. Microb..

[44] Wei Y. (2014). Coloning and Functional Identification of Laccase Gene (Lac1) in Pathogenicity from Colletotrichum gloeosporioides the Pathogen of Mango Anthracnose Disease.

[45] Luo C., He X.H., Chen H., Hu Y., Ou U.S. (2013). Molecular cloning and expression analysis of four actin genes (*MiACT*) from mango. Biol. Plant..

[46] Zhao Z., Gao A., Chen Y., Huang J., Dang Z., Luo R. (2015). Cloning and Sequence Analysis of *PAL* Gene in Mango. J. Anhui Agric. Univ..

[47] Wang P., Luo Y., Huang J., Gao S., Zhu G., Dang Z., Gai J., Yang M., Zhu M., Zhang H. (2020). The genome evolution and domestication of tropical fruit mango. Genome Biol..

[48] Lu D. (2020). Study on the Biological Function of Type II Myosin Regulating Light Chain MoRlc1 of Magnaporthe grisea.

[49] Cañero D.C., Roncero M.I. (2008). Functional analyses of laccase genes from *Fusarium oxysporum*. Phytopathology.

[50] Lǚ Z., Kang X., Xiang Z., He N. (2017). Laccase Gene *Shlac* Is Involved in the Growth and Melanin Biosynthesis of *Scleromitrula shiraiana*. Phytopathology.

[51] Liu X., Liu J., Yin Z., Zhang H., Zhang Z. (2020). Research progress on early infection mechanism of interaction between *Magnaporthe oryzae* and rice. Bul. Natl. Nat. Sci. Found China.

[52] Wei Y., Liu X., Zhang H., Zhang X., Qi Y., Xie Y., Lu Y., Cao S., Pu J. (2013). Cloning and sequence analysis of *Lac1* gene of *Colletotrichum gloeosporioides*. J. Fruit Tree.

[53] Liu S., Wang Q., Liu N., Luo H., He C., An B. (2022). The histone deacetylase *HOS2* controls pathogenicity through regulation of melanin biosynthesis and appressorium formation in *Colletotrichum gloeosporioides*. Phytopathol. Res..

[54] Li X., Ke Z., Xu S., Tang W., Liu Z. (2021). The G-protein alpha subunit *CgGa1* mediates growth, sporulation, penetration and pathogenicity in *Colletotrichum gloeosporioides*. Microb. Pathog..

[55] Mushtaq A., Tariq M., Ahmed M., Zhou Z., Ali I., Mahmood R.T. (2021). Carbamoyl phosphate synthase subunit *CgCPS1* is necessary for virulence and to regulate stress tolerance in *Colletotrichum gloeosporioides*. Plant Pathol. J..

[56] Li X., Liu S., Zhang N., Liu Z. (2021). Function and transcriptome analysis of an oligopeptide transporter *CgOPT2* in the rubber anthracnose fungus *Colletotrichum gloeosporioides*. Physiol. Mol. Plant Pathol..

[57] Wang X., Lu D., Tian C. (2021). *CgEnd3* regulates endocytosis, appressorium formation, and virulence in the poplar anthracnose fungus *Colletotrichum gloeosporioides*. Int. J. Mol. Sci..

[58] Duan Z.B., Qiang G., Lu D.D., Shi S.H., Wang C.S. (2009). Appressorial differentiation and its association with cAMP in the insect pathogenic fungus *Metarhizium anisopliae*. Mycosystema.

[59] Wang C.S., Typas M.A., Butt T.M. (2002). Detection and characterisation of *pr1* virulent gene deficiencies in the insect pathogenic fungus *Metarhizium anisopliae*. FEMS Microbiol. Lett..

[60] Xu X., Wang C., Li H., Shang W., Hu X. (2018). Cloning and functional analysis of *VdCPMO* gene from *Rotaea dahlia*. J. Mycol..

[61] Ahamedemujtaba V., Atheena P.V., Bhat A.I., Krishnamurthy K.S., Srinivasan V. (2021). Symptoms of piper yellow mottle virus in black pepper as influenced by temperature and relative humidity. Virus Dis..

