# Supplementary material for: The Involvement of the Laccase Gene Cglac13 in Mycelial Growth, Germ Tube Development, and the Pathogenicity of Colletotrichum gloeosporioides from Mangoes
Source: J Fungi (Basel). 2023 Apr 23;9(5):503. doi: 10.3390/jof9050503 (PMC10219046; doi:10.3390/jof9050503)
Supplement: Supplementary file 1 [file jof-09-00503-s001.zip › jof-2281340-supplementary.pdf]

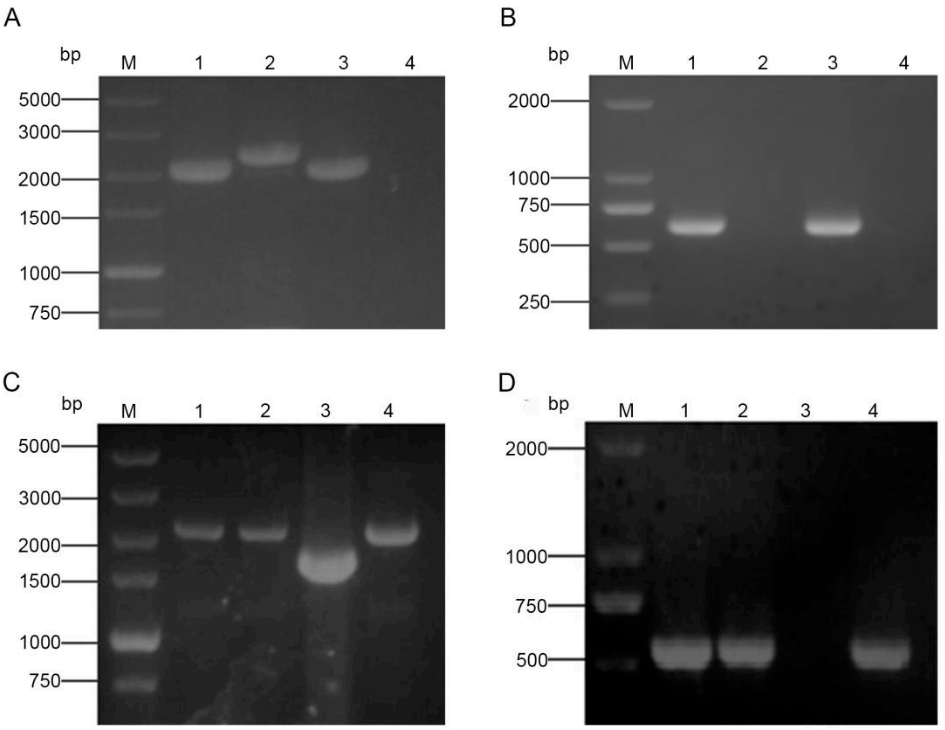

Figure S1 The electropherogram of primers F1/R1, F2/R2, F3/R3 and F4/R4.

A: the electrophoretogram of F1/R1. Lane 1: mutant *ΔCglac13H*, lane 2: wild-type, lane 3: pCglac13H, lane 4: ddH_2O; B: the electrophoretogram of F2/R2. Lane 1: mutant *ΔCglac13H*, lane 2: wild-type, lane 3: pCglac13H, lane 4: ddH_2O; C: the electrophoretogram of F3/R3. Lane 1-2: the complementary strain *C-ΔCglac13H*, lane 3: wild-type, lane 4: pC-ΔCglac13H; D: the electrophoretogram of F4/R4. Lane 1-2: the complementary strain *C-ΔCglac13H*, lane 3: wild-type, lane 4: pC-ΔCglac13H.

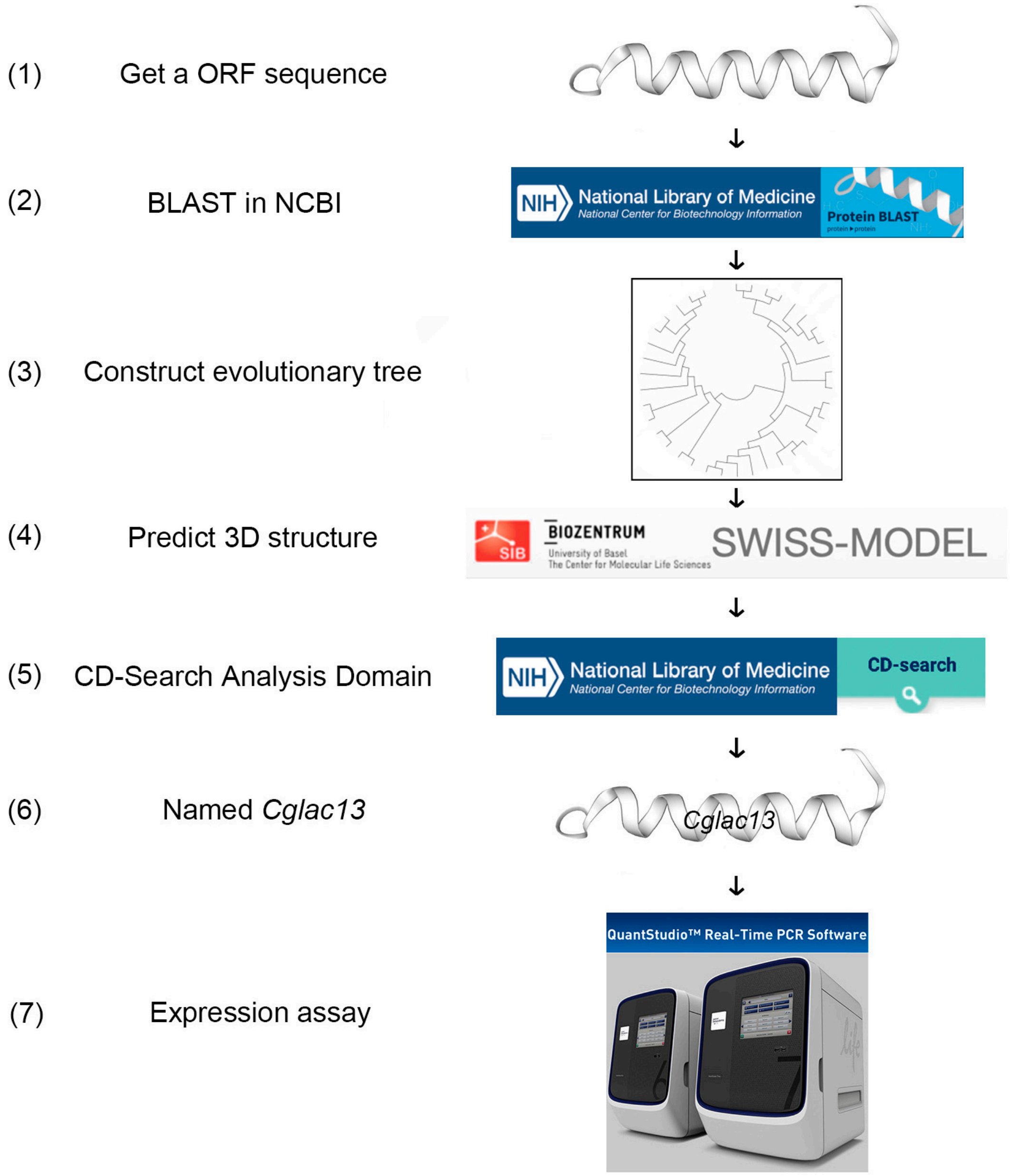

Figure S2 The flowchart of Cglac13 identification.

Text S1 Primer sequence of *Cglac13*

Primer name	Sequence (5′–3′)	Expected length /bp
Cglac13–qPCR–F	CCACTGCCACAATCTTAT	178
Cglac13–qPCR–R	TTGCACCTTCTGCACAAC	
5Cglac13–MHF	AACGAAAGCCAGCGACAA	445
5Cglac13–MHR	CTGTACTCAGGACTCAGCCAGT	
3Cglac13–MHF	ATGGAGCAGGTTGATGAGATT	458
3Cglac13–MHR	ATGGGAAGGAGGAGTGGG	
hygB–F	AACTGGTTCCCGGTCGGC	1412
hygB–R	AACTGATATTGAAGGAGCATTTTTT	
5Cglac13–CF	TCTAGACAGACACGCAGC	2140
5Cglac13–CR	AAATCTTACACCCCCAAT	
3Cglac13–CF	TAAGATTACGGTGATGGC	260
3Cglac13–CR	GATAGATTCTGATGGGGA	
F1–Cglac13H	CGGGAGCACAACAGCAAT	2199 (Mutant)<br>2554 (Wild–type)<br>2199 (pCglac13H)
R1–Cglac13H	GGAGGAGTGGGTTAGCGTAG	
F2–H850	TTGTCCGTCAGGACATTGTT	610 (Mutant)<br>0 (Wild–type)<br>610 (pCglac13H)
R2–H852	AACTCACCGCGACGTCTGTC	
F3–Cglac13 BAR	GAGTGCCCGCTCCTGTGGTA	2169 (Complementary strain)<br>1618 (Wild–type)<br>0 (Mutant)
R3–Cglac13 BAR	GATTCTGATGGGGAAATTTG	
F4–Bar	TCAAATCTCGGTGACG	552 (Complementary strain)<br>0 (Mutant; Wild–type)
R4–Bar	ATGAGCCCAGAACGACGC	
MiActin–qPCR–F	GTTTCCCAGTATTGTGGGTAGG	134
MiActin–qPCR–R	AGATCTTTTCCATATCATCCCAGTT	
MiPAL–qPCR–F	GTCGCATAGGAGAACGAAGC	204
MiPAL–qPCR–R	AACTTGGTGATGGCTTCCAG	
Mi4CL–qPCR–F	GAATACGCTTTCTCTCTCAGAG	188
Mi4CL–qPCR–R	GAGTTGGGAGAGAGTACAAATG	
MiCAD–qPCR–F	CGGCAAGATTACACCTTACACAT	170
MiCAD–qPCR–R	TAACCACCCCAGTAATTTCATGC	
MiCOMT–qPCR–F	GGCAAAGATCCCAGATTCAA	228
MiCOMT–qPCR–R	CAAAGATGGAGCATCGTCAA	

Cglac13 **Wild-type**

GGTCGGCTTCTGCGCACGCCAGCTGAACCGACTCGTCGGGTCGCTTGTTCGCCGTGCGTCTATTC
GTGCCAAACTTGGCGATTGCTTGACTCGCCGAGAGATCAAGACTGCCCCTAAACCTCGCGGGGA
TGCACAGTTTCTGCAGGAACGGCTGGGTTTAGGATGCGCTGACTGCCAAGGGATCATCCGATCA
CTGACGACCGCCAACGAAAGCCAGCGACAAAAGCCCAGATTCTCTCATCTTGTAGAAAAGCTG
CAGTCTGAGCCATCACGCAGAATGGCTTCGTTTGCATGCACGTTCGGACTGCTAAGGCCGGGAG
CACAACAGCAATAGTCTAGACAGACACGCAGCGCCACTCGGGTGATTATTGGTGTTATACGAAC
TTGCTGAGGATCTGGATGAGGCTCCGCGCGGGGGGAAGGGGGCTCTCGTCAGACGTATATAGAT
CCGCAATTGCCATCTCTCGTATTACATGTTCCGGTTGTTCGCCAGTGAAGTTTTGTTTTGTGACGA
ACAAGGTCCTACTTCTCTTGTCTCTTCGATCTTTACCGACCCCGTCCAAAATGCTCTTCCGGGCTT
CTCACCTCGTGACCGCGATGGCCCTTTTCGAAGGGTCGTCAGCGAAGGACTGGCTGAGTCCTGA
GTACAGGTTCTTTTACCAGTTCCCGTTGCCCATCCCGCCGGTGAAGCAGCCCAAGATGTGAGTTT
CTAATGAAATGACAAGTGATGTGGACCAATTCTGACATTTTGTAGGTCAATCACCAACCCGGTCA
CCAACAAGCAGATTGACTACTACGAGGTCGACATCGTGCCCTTCAAGCAGCAGGTCTACCCGAA
CAAGGGACCGGCTTCGCTCGTTGGCTATGATGGACTTTCACCCGGCCCCACGTTTCTCGTCCCCA
GAGATCGAGAGACGGTTGTGAGATTCACCAACAAGGCCACGCTGCCAAGCGCCGTCCATTTGC
ATGGATCGCCTTCTGTAGGATAACCCACTTCAGAGTTGAGATTCAATATTTGGGGCTAACATTCCT
GAAGAGGGCCCCTTGGGACGGTTGGGCCGAAGACAAGATCCAGCCTGGAGAGTACAAGGACTA
TTATTACCCCAACAGCCAGAGTGCCCGCTCCTTGTGGTATCACGACCACGCCATGGACATTGTAA
GACTCCCTCACACCACCTTTTAGCCAAGCTAACTCATCATCACAGACCGCAGTCAACGCCTACTT
TGGCCAGGCCGGCGCGTACATCGTCCAAGACCCAGCCGAAGACGCACTCGGTCTCCCGACGGG
CTACGGCGTCAACGACATCCCCCTCGTCCTGTCCTCGAAGCAGTACAACGCCGACGGCTCCCTC
TTCTCGCCCGCCGGCGAGACCGACAGTCTCTGGGGAGACGTGATCCACGTCAACGGCCAGCCG
TGGCCCTTCTTCAAGGTCGAGCCGCGCAAGTACCGCCTGCGCTTCCTCAACGCCGCCGTCTCGC
GCTCTTTCATCCTCTACTTCAGGCGCCAGACCGGCGGTGCCAACATCCCGTTTCAAGTCATTGCA
TCTGACGCCGGACTGCTCACTGGCCCGATCACCACGAGCACACTCACCATGTCTATGGCGGAGC
GATGGGAGGTCGTGGTCGACTTTTCCGGATACGCGGGGCAGAACATCTCACTTCTAAACCAGAA
AGACGTCGGCAAGGACACAGACTACGGCTTCACGGACCAAGTAATGCGCTTCATCGTGGGCAA
GTCCGCCGCCGCCGCAGACACCTCCCTCGTCCCCGCCAAACTCCGCAACGTGCCCTTCCCGCCG
CGGCAGGGCGACGGCGTCGACCGCCGTTTCAAGTTCCACCGGTCCAACGGCGAGTGGCAGATC
AACGGCGTCGGCTTCAGCGACGTGGCGAACCGCGTGCTAGCCCGCGTCCCGCGTGGCACCGTC
GAGATCTGGGAGTTCGAGAACGGCGGCGGCGGCTGGACGCACCCGATCCACGTGCACCTCGTC
GACTTCCGTATTCTCAGCCGGACCAAGGCGAAGCGCCCTGTGCTCGCGTACGAGGCTGCTGGGT
TAAAGGACGTGGTGTGGCTGGATGCCGGGGAGGTCGTCCGCGTGGAGGCGCACTATGCGCCGTG
GGACGGGGTGTACATGTTCCACTGCCACAATCTTATCCACGAGGACCACGAGATGATGGCGGCG
TTTAATGTGACGGCGCTGGCGGACTTGGGGTATACTGAGACGTCTTTTGCGGACCCGATGGAGGC
AAGATGGAGGGCGGTGCCGGTCTCGGCGGCGGCGTTTCAGGCGGATGCGGTTGTGCAGAAGGT
GCAATCGATGGCGGCACTGCAGCCGTATAATAATGAGGCGGAGGTGCTGCAGAGGCTGGACCA
GTATTGGGCTGCCCGTGGTGGTGTGAAGATGAGGCGCGGCAAGAGGATGGAGCAGGTTGATGA
GATTGAGGGGTGATGTTGCTGAATTGGGGGTGTAAGATTTGTAAGATTACGGTGATGGCATCTTC
ATTTATACATATTACAACAAGTGTACTCTAGATTTCTTAAGCTACTTGTATTAAAACAACGAAGCA
TTCTTCTAGTAATGTTTTGAAGATGAACTAGGTTTCTTTGACCACAACTCGAAAGAGTAACTTCGT
GGGGCATAGTGAAGGCTTGCAACGAATAGATTTGAATTTCAAGTTCAAGGAGCTTTCACCTCAA
ATGAAGGTATAAGTCGCAAATTTCCCCATCAGAATCTATCTTGTGACGAACTCTGATGCAAGTCT

ATTTCTTTTGATGTCAAGTCTTTCGTGATCTATGCGCAAAAGTCCTTCACAACGCTGGTCATCACG
GCAGTAACCTCGCGAAGACTCTCTACGCTAACCCACTCCTCCTTCCCATGAAGTCCAAAACCCTT
TGGTCCGTACACAATCGACGGGATACCGACCTGATGTAATAGCGCGGCATCGCACCAGAACGGC
ATGCCATTCGGCTCAGGAACACTGCCGGTGACTCTCGACACGTGGGAGACAAAGTCTGTGAAA
AATTCATCGTCCGCAGCTAGCGCGAAACAGGGGCGCTCAAATGTCACACGCGGCGGGGCGTAC
TTGAACTCTGGCGTGCTCGATGCTATCCCCGCGAGAATTCCTTCCACATCGCGCAGGATGGACTC
CCTCGACTGCGACGGAACGGTGCGAAACTCAACAGTCAGTGTGCACTTGTCCGGATAACTCGA
GGGCTCCTCGCCGCCTTGGATGAGCCCGCCATGCAATGACGCCTTGCCGAGACGGTCGTCCGTC
GGCAGCCCCTTTGCATACCCCAGACGGCAGTCTGCACGCCCCAGCAGCAGATGGCATCAACTCC
CTTATCCGGCATGGACCCGTGAGCCGCAACGCCGAGCACGTCATCTCACCCCATATAGAGACAC
AGTGC

Primer name	Sequence (5′–3′)	Expected length /bp
Cglac13–qPCR–F	CCACTGCCACAATCTTAT	178
Cglac13–qPCR–R	TTGCACCTTCTGCACAAC	
5Cglac13–MHF	AACGAAAGCCAGCGACAA	445
5Cglac13–MHR	CTGTACTCAGGACTCAGCCAGT	
3Cglac13–MHF	ATGGAGCAGGTTGATGAGATT	458
3Cglac13–MHR	ATGGGAAGGAGGAGTGGG	
hygB–F	AACTGGTTCCCGGTCGGC	0
hygB–R	AACTGATATTGAAGGAGCATTTTTT	
5Cglac13–CF	TCTAGACAGACACGCAGC	2140
5Cglac13–CR	AAATCTTACACCCCCAAT	
3Cglac13–CF	TAAGATTACGGTGATGGC	260
3Cglac13–CR	GATAGATTCTGATGGGGA	
F1–Cglac13H	CGGGAGCACAACAGCAAT	2554
R1–Cglac13H	GGAGGAGTGGGTTAGCGTAG	
F2–H850	TTGTCCGTCAGGACATTGTT	0
R2–H852	AACTCACCGCGACGTCTGTC	
F3–Cglac13 BAR	GAGTGCCCGCTCCTGTGGTA	1618
R3–Cglac13 BAR	GATTCTGATGGGGAAATTTG	
F4–Bar	TCAAATCTCGGTGACG	0
R4–Bar	ATGAGCCCAGAACGACGC	

ΔCglac13H **Mutant**

GGTCGGCTTCTGCGCACGCCAGCTGAACCGACTCGTCGGGTCGCTTGTTCGCCGTGCGTCTATTC
GTGCCAAACTTGGCGATTGCTTGACTCGCCGAGAGATCAAGACTGCCCCTAAACCTCGCGGGGA
TGCACAGTTTCTGCAGGAACGGCTGGGTTTAGGATGCGCTGACTGCCAAGGGATCATCCGATCA
CTGACGACCGCCAACGAAAGCCAGCGACAAAAGCCCAGATTCTCTCATCTTGTAGAAAAGCTG
CAGTCTGAGCCATCACGCAGAATGGCTTCGTTTGCATGCACGTTCGGACTGCTAAGGCCGGGAG
CACAACAGCAATAGTCTAGACAGACACGCAGCGCCACTCGGGTGATTATTGGTGTTATACGAAC
TTGCTGAGGATCTGGATGAGGCTCCGCGCGGGGGGAAGGGGGCTCTCGTCAGACGTATATAGAT
CCGCAATTGCCATCTCTCGTATTACATGTTCCGGTTGTTCGCCAGTGAAGTTTTGTTTTGTGACGA
ACAAGGTCCTACTTCTCTTGTCTCTTCGATCTTTACCGACCCCGTCCAAAATGCTCTTCCGGGCTT
CTCACCTCGTGACCGCGATGGCCCTTTTCGAAGGGTCGTCAGCGAAGGACTGGCTGAGTCCTGA
GTACAGAACTGGTTCCCGGTCGGCATCTACTCTATTCCTTTGCCCTCGGACGAGTGCTGGGGCGT
CGGTTTCCACTATCGGCGAGTACTTCTACACAGCCATCGGTCCAGACGGCCGCGCTTCTGCGGGC
GATTTGTGTACGCCCGACAGTCCCGGCTCCGGATCGGACGATTGCGTCGCATCGACCCTGCGCCC
AAGCTGCATCATCGAAATTGCCGTCAACCAAGCTCTGATAGAGTTGGTCAAGACCAATGCGGAG
CATATACGCCCGGAGGCGCGGCGATCCTGCAAGCTCCGGATGCCTCCGCTCGAAGTAGCGCGTC
TGCTGCTCCATACAAGCCAACCACGGCCTCCAGAAGAGGATGTTGGCGACCTCGTATTGGGAAT
CCCCGAACATCGCCTCGCTCCAGTCAATGACCGCTGTTATGCGGCCATTGTCCGTCAGGACATTG
TTGGAGCCGAAATCCGCATGCACGAGGTGCCGGACTTCGGGGCAGTCCTCGGCCCAAAGCATC
AGCTCATCGAGAGCCTGCGCGACGGACGCACTGACGGTGTCGTCCATCACAGTTTGCCAGTGAT
ACACATGGGGATCAGCAATCGCGCATATGAAATCACGCCATGTAGTGTATTGACCGATTCCTTGC
GGTCCGAATGGGCCGAACCCGCTCGTCTGGCTAAGATCGGCCGCAGCGATCGCATCCATGGCCT
CCGCGACCGGCTGGAGAACAGCGGGCAGTTCGGTTTCAGGCAGGTCTTGCAACGTGACACCCT
GTGCACGGCGGGAGATGCAATAGGTCAGGCTCTCGCTGAACTCCCCAATGTCAAGCACTTCCGG
AATCGGGAGCGCGGCCGATGCAAAGTGCCGATAAACATAACGATCTTTGTAGAAACCATCGGCG
CAGCTATTTACCCGCAGGACATATCCACGCCCTCCTACATCGAAGCTGAAAGCACGAGATTCTTC
GCCCTCCGAGAGCTGCATCAGGTCGGAGACGCTGTCGAACTTTTCGATCAGAAACTTCTCGACA
GACGTCGCGGTGAGTTCAGGCTTTTTCATTTGGATGCTTGGGTAGAATAGGTAAGTCAGATTGAA
TCTGAAATAAAGGGAGGAAGGGCGAACTTAAGAAGGTATGACCGGGTCGTCCACTTACCTTGCT
TGACAAACGCACCAAGTTATCGTGCACCAAGCAGCAGATGATAATAATGTCCTCGTTCCTGTCTG
CTAATAAGAGTCACACTTCGAGCGCCGCCGCTACTGCTACAAGTGGGGCTGATCTGACCAGTTG
CCTAAATGAACCATCTTGTCAAACGACACAAATTTTGTGCTCACCGCCTGGACGACTAAACCAA
AATAGGCATTCATTGTTGACCTCCACTAGCTCCAGCCAAGCCCAAAAAATGCTCCTTCAATATCA
GTTATGGAGCAGGTTGATGAGATTGAGGGGTGATGTTGCTGAATTGGGGGTGTAAGATTTGTAAG
ATTACGGTGATGGCATCTTCATTTATACATATTACAACAAGTGTACTCTAGATTTCTTAAGCTACTT
GTATTAAAACAACGAAGCATTCTTCTAGTAATGTTTTGAAGATGAACTAGGTTTCTTTGACCACA
ACTCGAAAGAGTAACTTCGTGGGGCATAGTGAAGGCTTGCAACGAATAGATTTGAATTTCAAGT
TCAAGGAGCTTTCACCTCAAATGAAGGTATAAGTCGCAAATTTCCCCATCAGAATCTATCTTGTG
ACGAACTCTGATGCAAGTCTATTTCTTTTGATGTCAAGTCTTTCGTGATCTATGCGCAAAAGTCCT
TCACAACGCTGGTCATCACGGCAGTAACCTCGCGAAGACTCTCTACGCTAACCCACTCCTCCTTC
CCATGAAGTCCAAAACCCTTTGGTCCGTACACAATCGACGGGATACCGACCTGATGTAATAGCG
CGGCATCGCACCAGAACGGCATGCCATTCGGCTCAGGAACACTGCCGGTGACTCTCGACACGTG
GGAGACAAAGTCTGTGAAAAATTCATCGTCCGCAGCTAGCGCGAAACAGGGGCGCTCAAATGT
CACACGCGGCGGGGCGTACTTGAACTCTGGCGTGCTCGATGCTATCCCCGCGAGAATTCCTTCCA

CATCGCGCAGGATGGACTCCCTCGACTGCGACGGAACGGTGCGAAACTCAACAGTCAGTGTGC ACTTGTCCGGATAACTCGAGGGCTCCTCGCCGCCTTGGATGAGCCCGCCATGCAATGACGCCTTG CCGAGACGGTCGTCCGTCGGCAGCCCCTTTGCATACCCCAGACGGCAGTCTGCACGCCCCAGCA GCAGATGGCATCAACTCCCTTATCCGGCATGGACCCGTGAGCCGCAACGCCGAGCACGTCATCT CACCCCATATAGAGACACAGTGC

5Cglac13-MHF	AACGAAAGCCAGCGACAA	445
5Cglac13-MHR	CTGTACTCAGGACTCAGCCAGT	
3Cglac13-MHF	ATGGAGCAGGTTGATGAGATT	458
3Cglac13-MHR	ATGGGAAGGAGGAGTGGG	
hygB-F	AACTGGTTCCCGGTCGGC	1412
hygB-R	AACTGATATTGAAGGAGCATTTTTT	
5Cglac13-CF	TCTAGACAGACACGCAGC	1785
5Cglac13-CR	AAATCTTACACCCCCAAT	
3Cglac13-CF	TAAGATTACGGTGATGGC	260
3Cglac13-CR	GATAGATTCTGATGGGGA	
F1-Cglac13H	CGGGAGCACAACAGCAAT	2199
R1-Cglac13H	GGAGGAGTGGGTTAGCGTAG	
F2-H850	TTGTCCGTCAGGACATTGTT	610
R2-H852	AACTCACCGCGACGTCTGTC	
F3-Cglac13 BAR	GAGTGCCCGCTCCTGTGGTA	0
R3-Cglac13 BAR	GATTCTGATGGGGAAATTTG	
F4-Bar	TCAAATCTCGGTGACG	0
R4-Bar	ATGAGCCCAGAACGACGC	

C-ΔCglac13H **Complementary strain**

GGTCGGCTTCTGCGCACGCCAGCTGAACCGACTCGTCGGGTCGCTTGTTCGCCGTGCGTCTATTC
GTGCCAAACTTGGCGATTGCTTGACTCGCCGAGAGATCAAGACTGCCCCTAAACCTCGCGGGGA
TGCACAGTTTCTGCAGGAACGGCTGGGTTTAGGATGCGCTGACTGCCAAGGGATCATCCGATCA
CTGACGACCGCCAACGAAAGCCAGCGACAAAAGCCCAGATTCTCTCATCTTGTAGAAAAGCTG
CAGTCTGAGCCATCACGCAGAATGGCTTCGTTTGCATGCACGTTCGGACTGCTAAGGCCGGGAG
CACAACAGCAATAGTCTAGACAGACACGCAGCGCCACTCGGGTGATTATTGGTGTTATACGAAC
TTGCTGAGGATCTGGATGAGGCTCCGCGCGGGGGGAAGGGGGCTCTCGTCAGACGTATATAGAT
CCGCAATTGCCATCTCTCGTATTACATGTTCCGGTTGTTCGCCAGTGAAGTTTTGTTTTGTGACGA
ACAAGGTCCTACTTCTCTTGTCTCTTCGATCTTTACCGACCCCGTCCAAAATGCTCTTCCGGGCTT
CTCACCTCGTGACCGCGATGGCCCTTTTCGAAGGGTCGTCAGCGAAGGACTGGCTGAGTCCTGA
GTACAGGTTCTTTTACCAGTTCCCGTTGCCCATCCCGCCGGTGAAGCAGCCCAAGATGTGAGTTT
CTAATGAAATGACAAGTGATGTGGACCAATTCTGACATTTTGTAGGTCAATCACCAACCCGGTCA
CCAACAAGCAGATTGACTACTACGAGGTCGACATCGTGCCCTTCAAGCAGCAGGTCTACCCGAA
CAAGGGACCGGCTTCGCTCGTTGGCTATGATGGACTTTCACCCGGCCCCACGTTTCTCGTCCCCA
GAGATCGAGAGACGGTTGTGAGATTCACCAACAAGGCCACGCTGCCAAGCGCCGTCCATTTGC
ATGGATCGCCTTCTGTAGGATAACCCACTTCAGAGTTGAGATTCAATATTTGGGGCTAACATTCCT
GAAGAGGGCCCCTTGGGACGGTTGGGCCGAAGACAAGATCCAGCCTGGAGAGTACAAGGACTA
TTATTACCCCAACAGCCAGAGTGCCCGCTCCTTGTGGTATCACGACCACGCCATGGACATTGTAA
GACTCCCTCACACCACCTTTTAGCCAAGCTAACTCATCATCACAGACCGCAGTCAACGCCTACTT
TGGCCAGGCCGGCGCGTACATCGTCCAAGACCCAGCCGAAGACGCACTCGGTCTCCCGACGGG
CTACGGCGTCAACGACATCCCCCTCGTCCTGTCCTCGAAGCAGTACAACGCCGACGGCTCCCTC
TTCTCGCCCGCCGGCGAGACCGACAGTCTCTGGGGAGACGTGATCCACGTCAACGGCCAGCCG
TGGCCCTTCTTCAAGGTCGAGCCGCGCAAGTACCGCCTGCGCTTCCTCAACGCCGCCGTCTCGC
GCTCTTTCATCCTCTACTTCAGGCGCCAGACCGGCGGTGCCAACATCCCGTTTCAAGTCATTGCA
TCTGACGCCGGACTGCTCACTGGCCCGATCACCACGAGCACACTCACCATGTCTATGGCGGAGC
GATGGGAGGTCGTGGTCGACTTTTCCGGATACGCGGGGCAGAACATCTCACTTCTAAACCAGAA
AGACGTCGGCAAGGACACAGACTACGGCTTCACGGACCAAGTAATGCGCTTCATCGTGGGCAA
GTCCGCCGCCGCCGCAGACACCTCCCTCGTCCCCGCCAAACTCCGCAACGTGCCCTTCCCGCCG
CGGCAGGGCGACGGCGTCGACCGCCGTTTCAAGTTCCACCGGTCCAACGGCGAGTGGCAGATC
AACGGCGTCGGCTTCAGCGACGTGGCGAACCGCGTGCTAGCCCGCGTCCCGCGTGGCACCGTC
GAGATCTGGGAGTTCGAGAACGGCGGCGGCGGCTGGACGCACCCGATCCACGTGCACCTCGTC
GACTTCCGTATTCTCAGCCGGACCAAGGCGAAGCGCCCTGTGCTCGCGTACGAGGCTGCTGGGT
TAAAGGACGTGGTGTGGCTGGATGCCGGGGAGGTCGTCCGCGTGGAGGCGCACTATGCGCCGTG
GGACGGGGTGTACATGTTCCACTGCCACAATCTTATCCACGAGGACCACGAGATGATGGCGGCG
TTTAATGTGACGGCGCTGGCGGACTTGGGGTATACTGAGACGTCTTTTGCGGACCCGATGGAGGC
AAGATGGAGGGCGGTGCCGGTCTCGGCGGCGGCGTTTCAGGCGGATGCGGTTGTGCAGAAGGT
GCAATCGATGGCGGCACTGCAGCCGTATAATAATGAGGCGGAGGTGCTGCAGAGGCTGGACCA
GTATTGGGCTGCCCGTGGTGGTGTGAAGATGAGGCGCGGCAAGAGGATGGAGCAGGTTGATGA
GATTGAGGGGTGATGTTGCTGAATTGGGGGTGTAAGATTTTCAAATCTCGGTGACGGGCAGGAC
CGGACGGGGCGGTACCGGCAGGCTGAAGTCCAGCTGCCAGAAACCCACGTCATGCCAGTTCCC
GTGCTTGAAGCCGGCCGCCCGCAGCATGCCGCGGGGGGCATATCCGAGCGCCTCGTGCATGCGC
ACGCTCGGGTCGTTGGGCAGCCCGATGACAGCGACCACGCTCTTGAAGCCCTGTGCCTCCAGGG
ACTTCAGCAGGTGGGTGTAGAGCGTGGAGCCCAGTCCCGTCCGCTGGTGGCGGGGGGAGACGT

ACACGGTCGACTCGGCCGTCCAGTCGTAGGCGTTGCGTGCCTTCCAGGGGCCCGCGTAGGCGAT
GCCGGCGACCTCGCCGTCCACCTCGGCGACGAGCCAGGGATAGCGCTCCCGCAGACGGACGAG
GTCGTCCGTCCACTCCTGCGGTTCCTGCGGCTCGGTACGGAAGTTGACCGTGCTTGTCTCGATGT
AGTGGTTGACGATGGTGCAGACCGCCGGCATGTCCGCCTCGGTGGCACGGCGGATGTCGGCCGG
GCGTCGTTCTGGGCTCATTAAGATTACGGTGATGGCATCTTCATTTATACATATTACAACAAGTGT
ACTCTAGATTTCTTAAGCTACTTGTATTAAAACAACGAAGCATTCTTCTAGTAATGTTTTGAAGAT
GAACTAGGTTTCTTTGACCACAACTCGAAAGAGTAACTTCGTGGGGCATAGTGAAGGCTTGCAA
CGAATAGATTTGAATTTCAAGTTCAAGGAGCTTTCACCTCAAATGAAGGTATAAGTCGCAAATTT
CCCCATCAGAATCTATCTTGTGACGAACTCTGATGCAAGTCTATTTCTTTTGATGTCAAGTCTTTC
GTGATCTATGCGCAAAAGTCCTTCACAACGCTGGTCATCACGGCAGTAACCTCGCGAAGACTCT
CTACGCTAACCCACTCCTCCTTCCCATGAAGTCCAAAACCCTTTGGTCCGTACACAATCGACGGG
ATACCGACCTGATGTAATAGCGCGGCATCGCACCAGAACGGCATGCCATTCGGCTCAGGAACAC
TGCCGGTGACTCTCGACACGTGGGAGACAAAGTCTGTGAAAAATTCATCGTCCGCAGCTAGCGC
GAAACAGGGGCGCTCAAATGTCACACGCGGCGGGGCGTACTTGAACTCTGGCGTGCTCGATGCT
ATCCCCGCGAGAATTCCTTCCACATCGCGCAGGATGGACTCCCTCGACTGCGACGGAACGGTGC
GAAACTCAACAGTCAGTGTGCACTTGTCCGGATAACTCGAGGGCTCCTCGCCGCCTTGGATGAG
CCCGCCATGCAATGACGCCTTGCCGAGACGGTCGTCCGTCGGCAGCCCCTTTGCATACCCCAGA
CGGCAGTCTGCACGCCCCAGCAGCAGATGGCATCAACTCCCTTATCCGGCATGGACCCGTGAGC
CGCAACGCCGAGCACGTCATCTCACCCCATATAGAGACACAGTGC

Primer name	Sequence (5′–3′)	Expected length /bp
Cglac13–qPCR–F	CCACTGCCACAATCTTAT	178
Cglac13–qPCR–R	TTGCACCTTCTGCACAAC	
5Cglac13–MHF	AACGAAAGCCAGCGACAA	445
5Cglac13–MHR	CTGTACTCAGGACTCAGCCAGT	
3Cglac13–MHF	ATGGAGCAGGTTGATGAGATT	1009
3Cglac13–MHR	ATGGGAAGGAGGAGTGGG	
hygB–F	AACTGGTTCCCGGTCGGC	0
hygB–R	AACTGATATTGAAGGAGCATTTTTT	
5Cglac13–CF	TCTAGACAGACACGCAGC	2140
5Cglac13–CR	AAATCTTACACCCCCAAT	
3Cglac13–CF	TAAGATTACGGTGATGGC	260
3Cglac13–CR	GATAGATTCTGATGGGGA	
F1–Cglac13H	CGGGAGCACAACAGCAAT	3105
R1–Cglac13H	GGAGGAGTGGGTTAGCGTAG	
F2–H850	TTGTCCGTCAGGACATTGTT	0
R2–H852	AACTCACCGCGACGTCTGTC	
F3–Cglac13 BAR	GAGTGCCCGCTCCTGTGGTA	2169
R3–Cglac13 BAR	GATTCTGATGGGGAAATTTG	
F4–Bar	TCAAATCTCGGTGACG	552
R4–Bar	ATGAGCCCAGAACGACGC	

MiActin (JF737036.1)

ATGGCCGATGCTGAAGATATTCAGCCCCTTGTCTGTGACAATGGAACTGGGATGGTGAAGGCCG
GGTTTGCCGGTGATGATGCTCCCAGGGCAGTGTTTCCCAGTATTGTGGGTAGGCCAAGACACAC
CGGTGTCATGGTTGGGATGGGGCAGAAGGATGCCTACGTCGGTGATGAGGCCCAATCCAAAAG
AGGTATTCTTACTTTGAAATACCCCATCGAGCATGGTATTGTGAGCAACTGGGATGATATGGAAA
AGATCTGGCATCACACTTTCTACAATGAGCTTCGAGTTGCCCCTGAAGAGCACCCAGTGCTTCTT
ACTGAGGCACCCTTAAATCCCAAGGCTAACAGAGAGAAGATGACCCAAATTATGTTTGAAACAT
TCAATGTCCCTGCCATGTATGTTGCTATCCAGGCCGTTCTGTCCCTCTATGCCAGTGGTCGTACAA
CTGGTATTGTGCTGGATTCTGGTGATGGTGTGTCTCACACTGTGCCAATTTACGAGGGTTATGCTC
TCCCACATGCAATTCTTCGATTGGATCTTGCTGGCCGTGATCTTACTGATGCATTGATGAAGATTC
TTACTGAGAGAGGTTACATGCTCACCACCACTGCCGAACGGGAAATTGTCCGAGACATGAAGG
AGAAGCTTGCTTATGTTGCTCTGGACTATGAGCAGGAACTTGAGACTGCTAAGAGCAGCTCTTCT
GTTGAGAAGAACTACGAGCTTCCTGATGGCCAGGTCATCACCATTGGAGCTGAGAGATTCCGCT
GTCCAGAGGTCCTCTTCCAGCCATCACTCATTGGTATGGAAGCTGCTGGTATCCATGAGACCACC
TACAACTCAATCATGAAGTGCGATGTTGATATCAGAAAGGATCTCTATGGTAACATTGTGCTCAG
TGGTGGTTCAACCATGTTCCCTGGCATTGCCGACCGTATGAGCAAGGAAATCACTGCACTTGCTC
CAAGCAGCATGAAGATCAAGGTGGTAGCTCCACCAGAGAGAAAATACAGTGTCTGGATAGGAG
GTTCAATCCTTGCATCCCTCAGCACCTTCCAGCAGATGTGGATTTCAAAGGGGGAGTATGATGAG
TCGGGTCCATCCATTGTCCACAGGAAGTGCTTCTAA

MiPAL

CTGGCAAGGGTGCCAAATTCCACCAATGAATGAGATTGCGCCCCAACTCCTTTTCACTTTTCAAA
ATGGAGTTCTGTCACGACAATCGCAATGGAGGCTTGTGCACGAGCGACCCGTTGAATTGGAACT
TCGCGGCGGAGTCACTGAAAGGGAGCCACCTCGATGAAGTTAAACGCATGGTGGAGGAGTTCA
GAAAGCCGGTGGTGAAGCTCGGGGGGGAGACGTTGACGATAGGTCAAGTGACTGCGATTGCGA
GTCATGATTCTGGCGTCAGGGTGGAGCTCTCGGAGACGGCGAGGGCCGGCGTCCAGGCCAGCA
GCGACTGGGTGATGGAGAGCATGAAGAATGGGACGGATAGTTATGGAGTGACCACTGGCTTTGG
TGCGACGTCGCATAGGAGAACGAAGCAAGGTGGCGCCCTGCAGAAGGAGCTCATAAGGTTCTT
GAATGCTGGAATTTTCGGCAGTGGTACAGAATCCTGCCACACGTTGCCTCATTCTGCGACAAGA
GCGGCGATGTTGGTCAGGATCAACACCCTGTTACAAGGATACTCAGGCATCAGGTTTGATATCCT
GGAAGCCATCACCAAGTTCCTTAACCATAACATCACCCCTTGCTTGCCACTCCGGGGCACAATTA
CTGCATCGGGCGACCTGGTTCCATTATCCTACATTGTGGGGCTCTTGACAGGCAGGCCCAATTCG
AAGGCGGTAGGGCCCAACGGGCAAGTCCTGAACGCCAGTGCAGCCTTCCAACTGGCTGGTATC
AGTAGTGGATTCTTTGAGTTGCAACCTAAAGAAGGTCTTGCACTAGTGAATGGCACAGCAGTTG
GTTCCGGCTTAGCTTCAACGG7TCTTTTCGAGGCTAACATACTTGCAATTCTGTCAGAAGTTTTAT
CAGCAATTTTTGCAGAAGTTATGAATGGAAAACCTGAGTTTACTGACCACTTGACACATAAATTG
AAGCACCATCCAGGCCAAATTGAAGCCGCAGCTATCATGGAGCACATTTTAGCTGGTAGCGGCT
ATGTTAAAGCAGCTCAAAAGTTGCACGAAATTGATCCTCTTCAGAAGCCGAAACAAGACAGATA
TGCTCTTAGAACATCTCCTCAATGGCTAGGCCCTCAGATTGAAGTCATCCGTGCTGCAACCAAAA
TGATTGAGAGGGAGATAAACTCTGTGAACGACAATCCATTGATTGATGTTGCAAGGAGTAAGGC
GTTGCACGGAGGCAATTTCCAGGGGACCCCAATTGGTGTTTCAATGGACAACACACGTCTTGCC
ATTGCTTCCATTGGTAAACTCATGTTTGCACAATTCTCCGAGCTGGTTAATGACTTTTACAACAAT
GGATTGCCTTCGAACCTTTCTGGGGGTCGCAATCCAAGTTTAGATTATGGATTCAAGGGTGCTGA
AATCGCCATGGCATCGTACTGCTCTGAACTTCAATTCCTTGCTAATCCTGTCACCAATCATGTCCA

GAGCGCCGAGCAACACAACCAAGATGTGAACTCTTTAGGCCTCATTTCTTCAAGAAAAACAGCT
GAAGCTGTTGACATATTGAAGCTCATGTCCTCTACATACTTGGTTGCTCTATGCCAAGCCATTGAC
TTGAGGCATTTGGAGGAGAACTTAAAGAACACTGTGAAGAACACAGTAAGTCAAGTGGCCAAG
AGAGTCCTGACAATGGGATTCAATGGTGAACTTCACCCATCAAGATTCTGCGAAAAAGACTTGA
TCAAAGTTGTCGACAGGGAATACGTTTTCGCATACATAGATGACCCCTGCAGCGCAACCTATCCA
TTGATGCAAAAATTAAGGCAAGTTCTGGTCGAACATGCAATGGCCAACGGAGAGAGAGAGAAA
AAATCAAGCACTTCAATTTTCCAGAAAATTGGAGCCTTTGAGGAGGAACTAAAAACCCTGCTTC
CCAAGGAAGTCGAAAGCACAAGAATTGAAATCGAGAATGGAAATGCAGCTGTTCCAAACAAG
ATAAAGGAATGCAGGTCCTATCCATTATACAAATTCGTGAGAGAAGAACTTGGAGCAAGTTTCCT
AACTGGTGAGAAAGTAAAGTCCCCAGGTGAGGAGTTTGACAAAGTTTTCTCGGCAATCTGTGCA
GGAAAGCTGATTGATCCAATGATGGAATGCTTAAAGGAGTGGAATGGAGCTCCTCTTCCTCTATG
CTAGGGGGGGATTAC

Mi4CL (XM_044609362)

ATGGAGCAGAGAAGTTTACATATTGATCCAAAGAGTGGTTTCAACTCAATAACAAAGACCTTCC
ACAGCCTCAGATCTCCCGTTCATCTTCTTCCCGAGAACGCTTTCCTTTCTGTTCCTGAATACGCTT
TCTCTCTCAGAGCCAAATCCCCGTGGCCTGATAATTCCATTGCTTTAGTCAACTCGATCACAGGTC
AACGGATTTCCTACTCTGAGTTCGGTCGCAGAACCAAGTCACTGGCCGCTTATCTACAGAAAGTA
ACTCAGCTTTCCAAATATGACGTAGCATTTGTACTCTCTCCCAACTCCATCCAAGTGCCAATCCTC
TACTTTTCTCTCCTCTCGCTCGGTGTTATCGTCTCTCCGGCAAATCCAGTGGCCACCAAATCTGAG
ATCTACGGGCAAATCCAGTTATCTAAGCCGGTAATAGCATTTGCCACGTCATCAACTGTCGACAA
GCTCCCAAAACTCAAACACCAGACGATCCTCATCGACTCTCCCGAATTCGAATCAATGATGGTA
AACTCAAAACATGAATTTGAGGAGGTTAAGGTGAGGCAATCTGATTTGGCAGCGATCATGTATTC
CTCTGGAACAACTGGAAAAGTTAAAGGTGTGATGTTGACGCACAAAAATTTAATAGCACAAAC
AGTAATAACCTCTGCAGCTTGGGAAATGTCTGAGAAGCGAGAATCCCCCTCAGTTATGCTTTTCA
CGCCGCCATATTTTCATATTTTTGGATTCTTCTATAGTATTCGATCGGTGGCTTTGAGTGAAAAAGT
GGTGGTGATGGAGAGGTTTGACCTGAAGAATATGTTGAAAGCTGTGCAGGAGTTTAGTGTTACA
CACGTGGCATTGACCCCGCCAGTAGTGGTGACTCTGTCGAAAGATGGATCCACCGACGGCTACG
ATTTGAGATCTCTTAAAACTGTACTGTGCGGCGCGGCTCCCCTTGGGAAGGAAGCAATCGCCGC
ATTTACAGCGAGATTTCCGAAAGTCATGTTGGTGCAGGGCTACGGGTTGACGGAAACGACGGCG
TCAGTTGCTCGGACTGTAGGTCCAGAAGAAAATAGGAACCGGGGCTCAACAGGGAAGCTTTCC
CCGGGTTTTGAGGCAAAAATCGTGGACCCTGAAACAACTGAAGCATTGCCTCCGTGCAAAGAA
GGGGAACTTTGGATTAGAGGACCTACAATAATGAAAGGATACGTTGGAGACCCAGAAGCAACTT
CGGCTACATTGGTATCTGATGGGTGGATGAGGACCGGAGACTTGTGCTACTTTGATAACAACGGT
TTCCTTTTTATTGTGGATAGACTCAAAGAATTAATCAAATATAAAGGATATCAGGTGGCTCCGGCG
GAACTAGAACAAGTGCTTATTTCCCACCCCCATGTGGCTGACGCAGCGGTTATACCGTATCCAGA
TAAAGAAACTGGTCAAGTTCCGATGGCTTTTGTGGTGAGACAGCCCCAGAGCTCTATCAGTGAG
GCCGAAATCATGGATTTTGTGGCGGAGCAGGTAGCGCCATACAAGAAGATAAGGCGTGTTGCAT
TTATCAATTCTATACCCAAAAGTGCAGCTGGCAAAATATTAAGAAAAGAGCTAATCAGGAAGGT
CGTTGTTGTTCCACCAGGGAGTTCATCCAGGTTGTAA

MiCAD (XM_044654805)

ATGGCTCAAACAACTCCGAACCATACCCAGACGGTGGCTGGCTGGGCTGCTCATGATTCGTCCG

GCAAGATTACACCTTACACATTCAAGCGAAGAGAGAACGGCGTCGAAGATGTGACCATAAAGA
TTATGTATTGTGGGATCTGCCATACCGATCTTCATCATGTGAGAAACGACTGGGGCATCACCATGT
ATCCTGTTGTTCCTGGGCATGAAATTACTGGGGTGGTTACGAAGGTTGGAAGCAACGTAAAGAA
TTTCAAGGTAGGAGATAAGGTGGGGGTTGGATGCTTGGCAGCATCTTGTTTGAAGTGTGAGTTCT
GCAAAAGCTCGCAAGAGAACTACTGTGATCAGCTCCAATTCGTGTATAATGGCATCTTCTGGGAT
GGCAGTATCACTTATGGCGGCTACTCTGAAATATTGGTCGCAGATCACAGGTACGTGGTGCACCT
ACCAGAGAATTTGTCAATGGCTTCTGTAGCGCCACTCTTGTGTGCGGGAATAACAGTGTTCACTC
CTTTGAAAGACAGCAATTTGATAGAGTCACCGGGGAAGAAGATCGGAGTGGTTGGTCTTGGGGG
TCTTGGACATGTGGCAATCAAATTTGGGAAGGCATTTGGTCATCATGTAACAGTCATCAGCACAT
CTCCATCAAAGGAAAAAGAAGCTAGAGACCTTTTGGATGCAGATGACTTCATCATCAGCACCAA
TGAAGAACAAATGAAGGCAGGCAGGAGAACCCTGGATTTTATACTGGACACAGTTGCGGCGAA
GCACTCTCTTGGCCCAATCTTAGAATTGCTCAAGGTGAATGGAACATTATCGTTTGTGGGTGCAC
CCGACAAGCCCGTTGAACTTCCTGCTTTTCCCTTGATTTTCGGCAAGAGAACTGTGAAGGGTAGC
ATAATAGGGGGAATTAAAGAGACACAGGAGATGATGGATCTGTGTGGCAAATACAACATCACAT
GCAACATTGAAGTCATAAAACCAGACCAAATTAACGAAGCCCTTGATCGCCTTGCAAGAAACG
ATGTCAGATACCGCTTTGTAATCGACATTGCCGGGAGTTTATCCAATATTCCTTAA

MiCOMT

ATGGGTTCCACAGGAGAAACTCAGATTACCCCAGTCCAAGTCTCAGATGAAGAAGAAGCTAATC
ACTTTGCCATGCAACTAACCAGCGCTTCAGTTCTACCCATGGTTCTCAAAGCCGCCATTGAACTT
GACCTTCTGGAGATCATTAAAAAAGCAGGCCCTGGAGCTTTTGTTTCTCCAAACGACATCGCATC
TCAGCTCCCCACAAAGAACCCAGATGCCCCCACCATGCTTGATCGCATTTTGCGCCTCCTCGCCA
GCTACTCTGTTCTTACTTGCTCTCTGCGTAATCTTCCTGATGGAAAAGTTGAGAGGCTTTATGGCC
TTGGAAAAGTCTGTAAATTTCTTACCAGAAATGAGGATGGCGTCAGTCTCGGTGATCTTTCTCTC
ATGAATCAAGATAAAATTTTGATGGAGAGCTGGTACTTCTTGAAAGATGCTGTGCTTGAAGGTGG
AATTCCATTTAACAAGGCCTATGGGATGACAGCATTTGAGTATCATGGCAAAGATCCCAGATTCA
ACAAGATCTTTAACAATGGAATGTCTTCACATTCGACAATCACCATGAAGAAAATTCTTGAGACT
TACAGAGGCTTCGAGGGTCTCAATTCTTTGGTTGATGTTGGTGGCGGAATTGGAGCCACACTCCA
CATGATTATCTCCAAATACCCTTTCATTAAAGGCATCAACTTTGATTTGCCTCATGTTATTGACGAT
GCTCCATCTTTGCCTGGCATCGAGCACGTTGGTGGAGACATGTTTGTTAGTGTACCAAAAGGAGA
CGCCATTTTCATGAAGTGGATATGTCACGATTGGAGCGATGAACACTGCTTGAAGTTCTTGAAGA
ACTGCTATGGAGCACTTCCAGATAATGGGAAAGTGATTGTTTGCGAATGTATTCTTCCAGTGATG
CCAGATCCAAGTCTTTCATCAAAGGTGACTATTCACATAGACTGTATAATGTTGGCTCATAATCCA
GGAGGAAAAGAGAGGACTGAACAAGAATTCAAGGCCTTGGCTAAAGATTCAGGCTTCCAAGGA
TTCCAAGTGGTTTGCTCTGCTTTCAACACTCACATTATGGAATTTATTAAGAGTGTTTGA
